# Prion-like transmission of human tau strains in the mouse brain

**DOI:** 10.1038/s41586-026-11061-x

**Published:** 2026-09-30

**Authors:** Sofia Lövestam, Aki Shimozawa, Airi Tarutani, Reiko Ohtani, Masami Masuda-Suzukake, Kazuko Hasegawa, Andrew C. Robinson, Yuko Saito, Shigeo Murayama, Mari Yoshida, Hisaomi Suzuki, Mitsumoto Onaya, Masato Hasegawa, Michel Goedert, Sjors H. W. Scheres

**Affiliations:** 1 MRC Laboratory of Molecular Biology, Cambridge, UK; 2 Laboratory of Neuropathology, Tokyo Metropolitan Institute of Medical Science, Tokyo, Japan; 3 Department of Clinical Medical Sciences, Tokyo Metropolitan Institute of Medical Science, Tokyo, Japan; 4 Division of Neurology, National Hospital Organization, Sagamihara National Hospital, Kanagawa, Japan; 5 Faculty of Biology, Medicine and Health, School of Biological Sciences, Division of Neuroscience, The University of Manchester, Manchester, UK; 6 Department of Neuropathology (The Brain Bank for Aging Research), Tokyo Metropolitan Institute for Geriatrics and Gerontology, Tokyo, Japan; 7 Department of Neurology, Graduate School of Medicine, Osaka Metropolitan University, Osaka, Japan; 8 Department of Neuropathology, Institute for Medical Science of Aging, Aichi Medical University, Aichi, Japan; 9 Department of Psychiatry, National Hospital Organization Shimofusa Psychiatric Center, Chiba, Japan

**Keywords:** Molecular neuroscience, Cryoelectron microscopy

## Abstract

Most neurodegenerative diseases are thought to spread through the brain by prion-like mechanisms, in which filamentous protein assemblies self-propagate by templated seeding^1^. Distinct conformations of amyloid filaments may provide the physical basis for the strains that lead to different diseases^2^. However, a central pillar of the prion hypothesis, that strains retain their structural identity upon transmission, has not been demonstrated. Here we show that the injection of tau filaments from the brains of individuals with Alzheimer’s disease or corticobasal degeneration into the brains of wild-type mice leads to the seeded assembly of amyloid filaments made of mouse tau with the same structures as those of the seeds. Thus, we show that, similar to prion strains, tau filaments propagate through templated seeding, and that the mouse is a suitable model to study the molecular mechanisms by which distinct tau folds drive disease-specific pathology in the brain.

## Abstract (web summary)

Mouse experiments show that Alzheimer’s disease and corticobasal degeneration tau filaments retain their distinct structures during templated seeding, supporting strain-specific tau propagation and disease modelling.

## Main

Misfolded filamentous aggregates of the prion protein cause neurodegeneration in diseases such as kuru, Creutzfeldt–Jakob disease (CJD), variant CJD, bovine spongiform encephalopathy, chronic wasting disease and scrapie^3,4^. Misfolded prion protein assemblies are believed to self-propagate by templated seeding, by which small amounts of filaments induce the misfolding of the native prion protein into filaments with the same conformation. This ability to self-propagate underlies the prion spread within tissues, between individuals of a given species and, depending on the prion protein sequence similarity, between individuals of distinct species. Various misfolded conformations of the prion protein give rise to prion strains that lead to different incubation times, lesion profiles and clinical phenotypes. Cryo-electron microscopy (cryo-EM) structures of prion filaments have shown that distinct amyloid folds provide the physical basis for prion strains^5–9^.

More than 20 neurodegenerative diseases are defined by the filamentous assembly of hyperphosphorylated tau^10^. Most disease cases with abundant tau filaments are sporadic, with Alzheimer’s disease (AD) being the most common tauopathy. Mutations in *MAPT*, the gene encoding tau, cause dominantly inherited forms of frontotemporal dementia with abundant tau inclusions, consistent with tau filament formation causing neurodegeneration. Cryo-EM structures of tau filaments from human brains have shown that distinct tau folds also characterize different diseases^11^ and that filaments with a single tau fold accumulate in the brains of individuals with a given tauopathy^12^. Moreover, small amounts of tau filaments can induce the assembly of normally disordered tau monomers into new filaments^13,14^.

Further support for the prion-like spreading of tau is provided by seeding animal models, in which tau filaments are injected into the mouse brain. Filaments are taken up by brain cells at the injection site, after which tau pathology spreads to distal regions, suggesting cell-to-cell propagation of tau filaments in the brain^14–19^. Seeding with human-brain-derived tau from different tauopathies recapitulates the cell-type-specific characteristics of those diseases^15^. Subsequent studies showed that these inclusions are robustly recapitulated in the wild-type mouse brain^17,20,21^ and in mice expressing all six human brain tau isoforms^22^. Moreover, the injection of AD tau seeds into mice with abundant amyloid-β plaques led to a marked enhancement of tau inclusions compared with injections into wild-type mice^23^. The injection of filaments assembled from recombinant tau into the brains of wild-type mice and of mice expressing all six human brain tau isoforms confirmed that tau filaments are the seeding species and showed that filaments with the AD-specific fold seed better than filaments with folds that have not been observed in human brains^24^.

However, it has so far not been shown that tau folds lead to distinct diseases by prion-like mechanisms and that conformational strains retain their identity on transmission. Human and mouse sequences are identical within the ordered cores of tau filaments from human brains, indicating that there may be no species barrier for the transmission of human disease-specific tau strains to the mice. But the atomic structures of tau filaments formed in the seeding mouse models described above remain unknown. To date, cryo-EM structures of tau filaments from transgenic mouse models have differed from those observed for filaments extracted from human brains^25,26^. Moreover, the intracerebral injection of recombinant wild-type α-synuclein filaments into the brains of mice transgenic for A53T human α-synuclein produced inclusions reminiscent of those in multiple system atrophy (MSA), but the structures of the recombinant filaments were distinct from those observed in MSA, and the seeded filaments only partially retained the structure of the seeds^27^.

Here, we report that intracerebral injection of tau filaments extracted from human brains of individuals with AD or corticobasal degeneration (CBD) into wild-type mice results in cell-type-specific tau inclusions characteristic of both diseases, and that these inclusions comprise filaments of endogenous mouse tau with the same structures as the seeds. Our results establish that distinct tau folds act as prion strains that propagate through templated seeding and drive disease-specific pathology in the brain.

## Cell-type-specific tau pathology

**Fig. 1:**
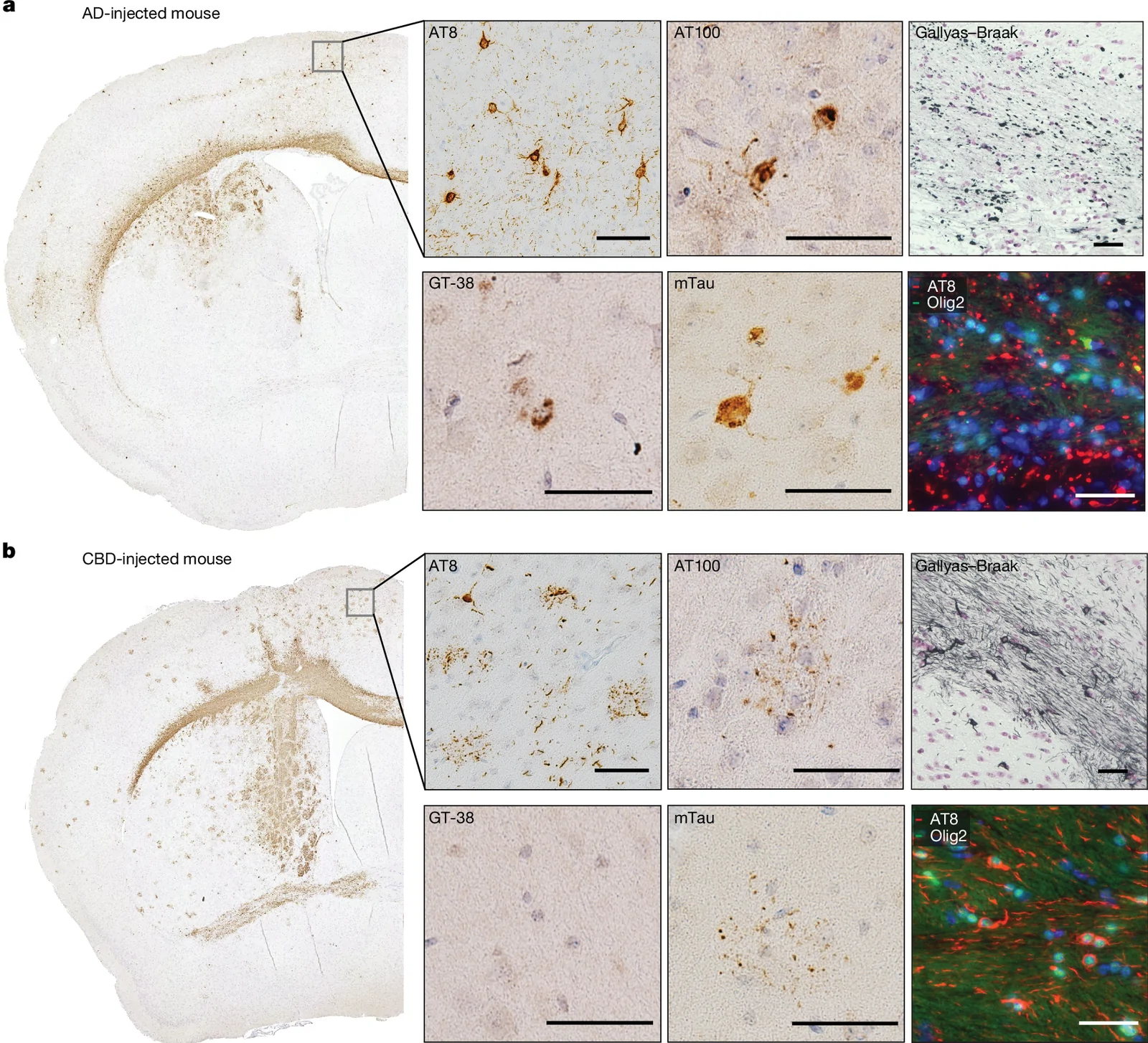
Histological staining of wild-type mouse brains 9 months after injection with human AD or CBD tau seeds. **a**,**b**, AD-injected (**a**) and CBD-injected (**b**) brains immunostained with antibodies AT8, AT100, mTau and GT-38; stained with Gallyas–Braak silver (black); and fluorescence double-immunostained with AT8 (red) and Olig2 (green). DAPI staining is shown in blue. Magnified images show the supplementary motor cortex on the dorsomedial surface of the frontal cortex. Fluorescence staining images show the corpus callosum. Scale bars, 100 µm (**a**,**b**).

**Extended Data Fig. 1:**
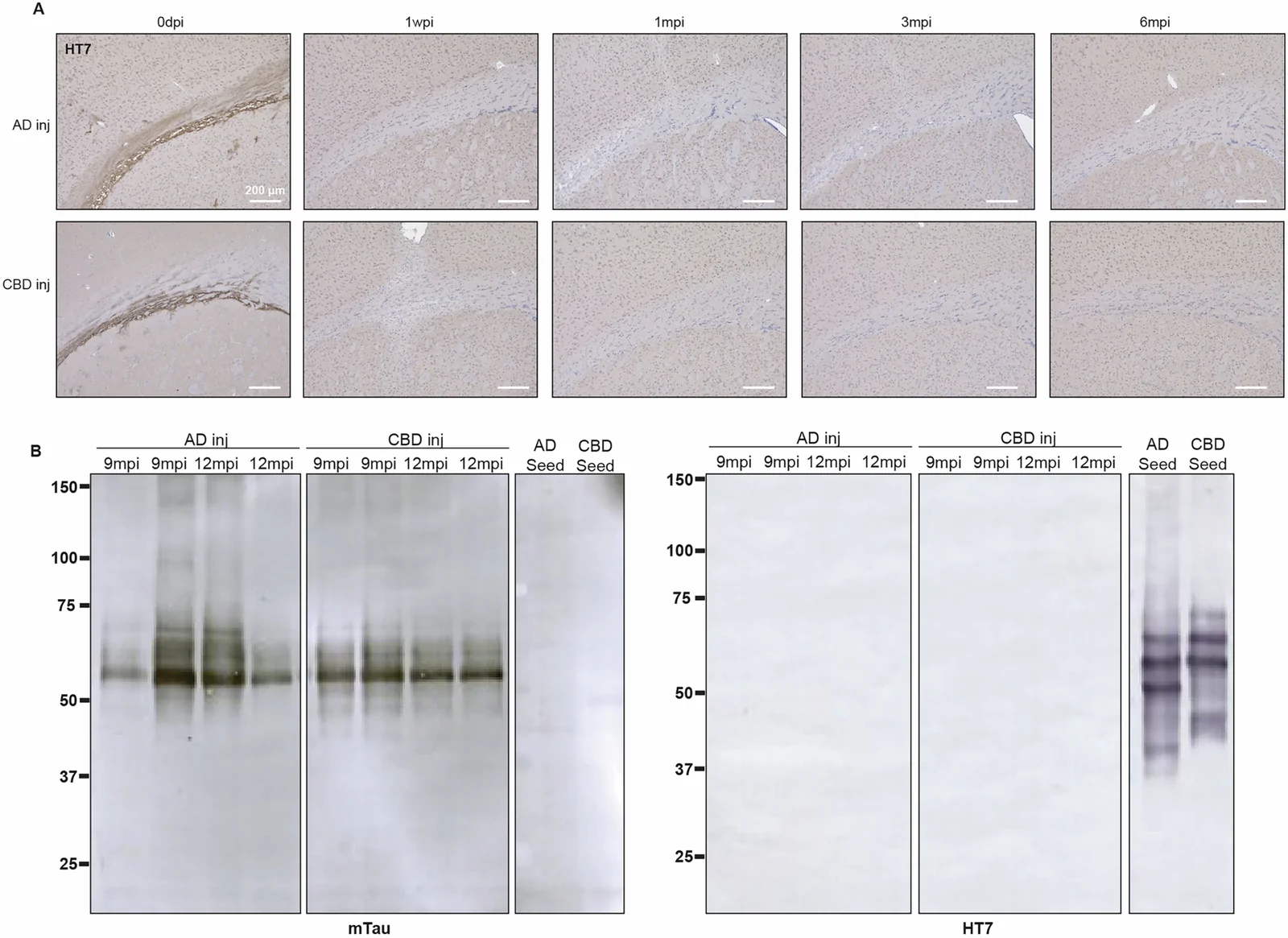
Immunohistochemistry and Western blotting of AD and CBD seed-injected mice 9 and 12 mpi. **A** Immunohistochemistry of the corpus callosum at the level of the frontal cortex with human tau-specific antibody HT7 after intrastriatal injection (0 dpi, 1 wpi, 1 mpi, 3 mpi and 6 mpi) of human AD or CBD tau seeds. Scale bar, 200 µm. **B**. Western blotting of sarkosyl-insoluble fractions from brain hemispheres of AD and CBD seed-injected mice at 9 mpi and 12 mpi and of the AD and CBD seeds that were used for injection with antibodies specific for mouse (mTau) or human (HT7) tau.

**Extended Data Fig. 2:**
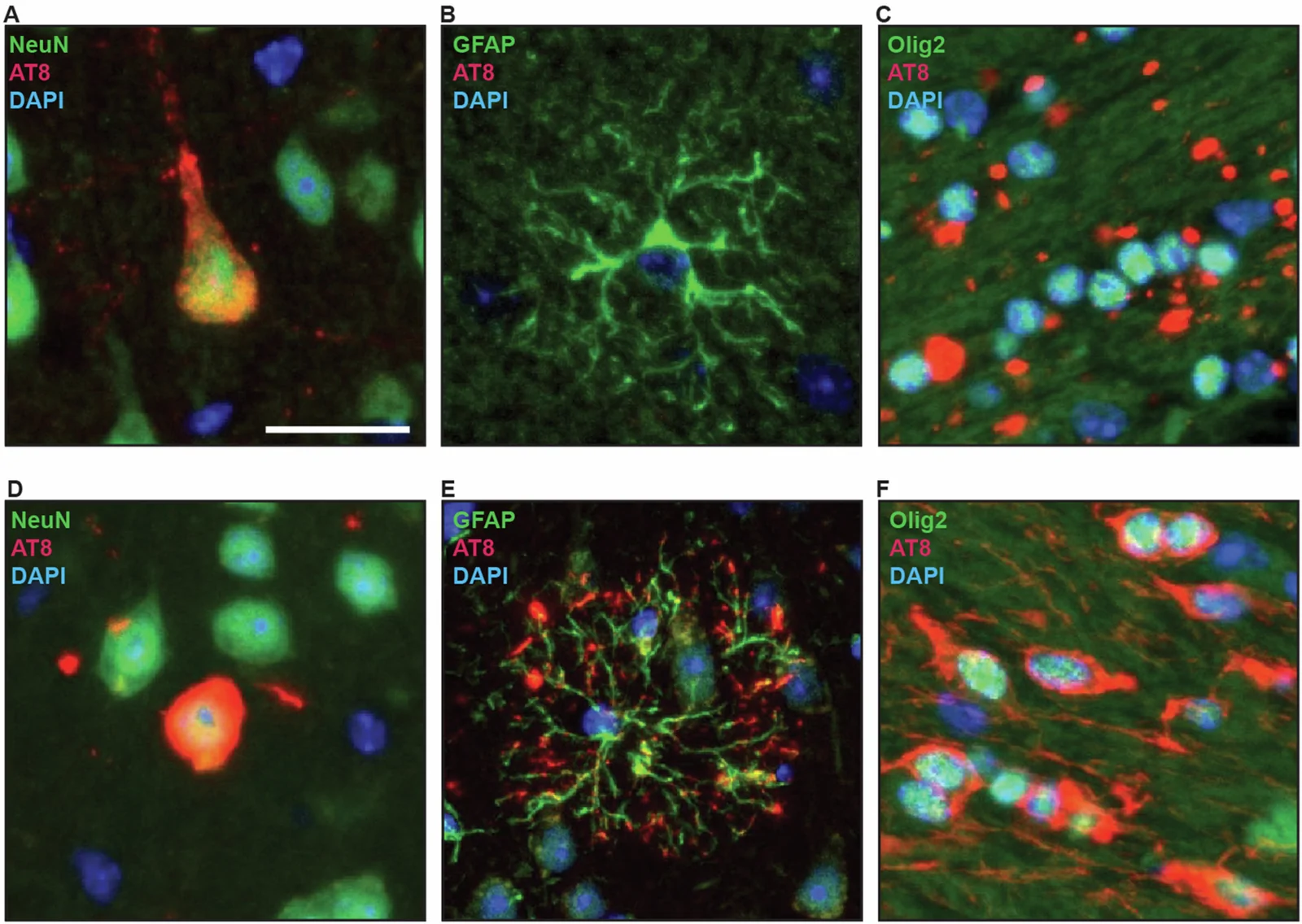
Double-labelling immunofluorescence of AD and CBD seed-injected mice 9 mpi. **A-C**, Corpus callosum at the level of the frontal cortex of AD seed-injected mice, and **D-F** for CBD seed-injected mice. NeuN staining marked neurons (A, D), GFAP staining marked astrocytes (B, E) and Olig2 staining marked oligodendrocytes (C, F). AT8 staining marked tau inclusions and DAPI staining marked nuclei, Scale bar in A (for A-F), 20 μm.

We injected tau seeds extracted from the frontal cortex of humans with AD or CBD into the striatum of 6–18-week-old wild-type mice (Fig. 1, Methods and Extended Data Tables 1–3). At 9 months post-injection (mpi), seeded tau pathology was detected using a mouse tau-specific antibody (mTau) and phosphorylation-dependent antibodies AT8 and AT100, indicating the accumulation of phosphorylated mouse tau. A human tau-specific antibody (HT7) gave no staining (Extended Data Fig. 1). AT8-positive tau pathology had spread from the injection site to the cerebral cortex, corpus callosum and other brain regions for both types of seeds, but the regional distribution and morphology of tau pathology differed depending on the seed (Extended Data Fig. 2). Mice injected with AD seeds showed tau pathology that was confined to neuronal cell bodies and their processes. By contrast, mice injected with CBD seeds showed tau pathology in both nerve cells and glial cells, forming coiled bodies and giving rise to plaque-like astrocytic inclusions, as has been observed in CBD in humans^28^. These findings indicate that tau seed structure influences both cellular targeting and pathological morphology in vivo.

**Extended Data Fig. 3:**
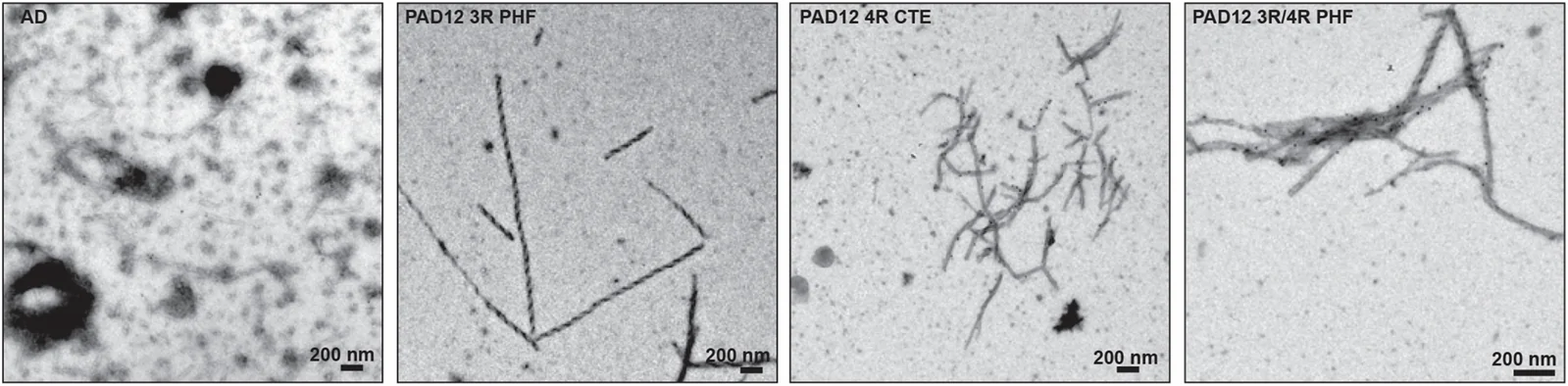
Negative-stain electron microscopy of tau filaments immunogold-labelled with the GT-38 antibody. From left to right: AD-derived filaments, recombinant PAD12 PHFs composed of 3R-only tau, recombinant PAD12 CTE filaments composed of 4R-only tau, and recombinant PAD12 PHFs composed of a mixture of 3 R and 4 R tau. Scale bar, 200 nm.

Staining with GT-38, an antibody that recognizes tau inclusions in AD but is negative in three-repeat (3R)-only or four-repeat (4R)-only tauopathies^29^, was positive in mice injected with AD seeds, but negative in those injected with CBD seeds. Immuno-EM with GT-38 was also negative for recombinant filaments with the AD fold made exclusively of 3R tau, but positive for filaments with the chronic traumatic encephalopathy (CTE) fold made exclusively of 4R tau or the AD fold consisting of a mixture of 3R and 4R tau (Extended Data Fig. 3). These findings indicate that the epitope of the GT-38 antibody lies within the second microtubule-binding repeat (R2), which is absent in 3R tau. Because mice express exclusively 4R tau in the adult brain, these results are consistent with the hypothesis that the epitope in R2 is accessible for GT-38 binding in the filaments from mouse brains injected with AD tau seeds, but that the same epitope is not accessible in the filaments from mice injected with CBD tau seeds, because it forms part of the ordered filament core.

## Phosphorylated mouse tau filaments

**Fig. 2:**
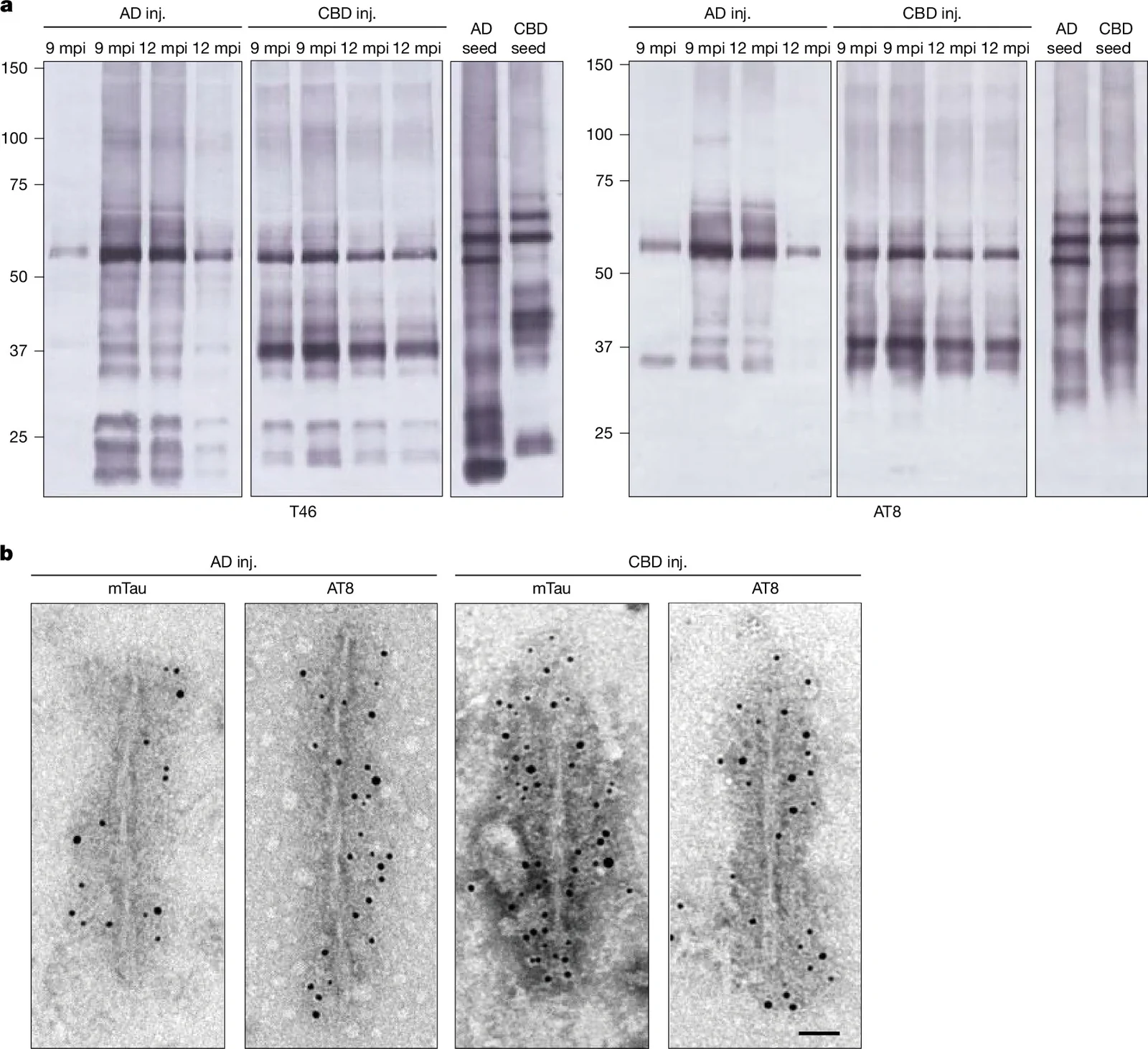
Biochemical characterization of seeded mouse tau. **a**, Western blots of sarkosyl-insoluble fractions from brain hemispheres using antibodies T46 and AT8 show accumulation of insoluble mouse tau at 9 mpi and 12 mpi for AD- and CBD-seed-injected wild-type mice. **b**, Negative-stain electron microscopy with immunogold labelling of AD- and CBD-injected mice 12 mpi with antibodies mTau and AT8. Scale bar, 25 nm (**b**).

Western blotting of sarkosyl-insoluble fractions from the injected mouse brains was positive for mTau and negative for HT7, suggesting that the insoluble fractions consisted only of mouse tau, in agreement with the immunohistochemistry described above. Distinct bands with the T46 and AT8 antibodies suggested biochemical differences between insoluble tau seeded with AD or CBD tau. Of note, seeding with CBD tau led to a 37 kDa band that is characteristic of CBD pathology (Fig. 2). Immuno-EM with mTau and AT8 antibodies of the sarkosyl-insoluble fractions confirmed that these fractions contained filaments composed of phosphorylated mouse tau. Tau filaments from mouse brains injected with CBD seeds had longer cross-over distances than those from brains injected with AD seeds (Fig. 2).

**Extended Data Fig. 4:**
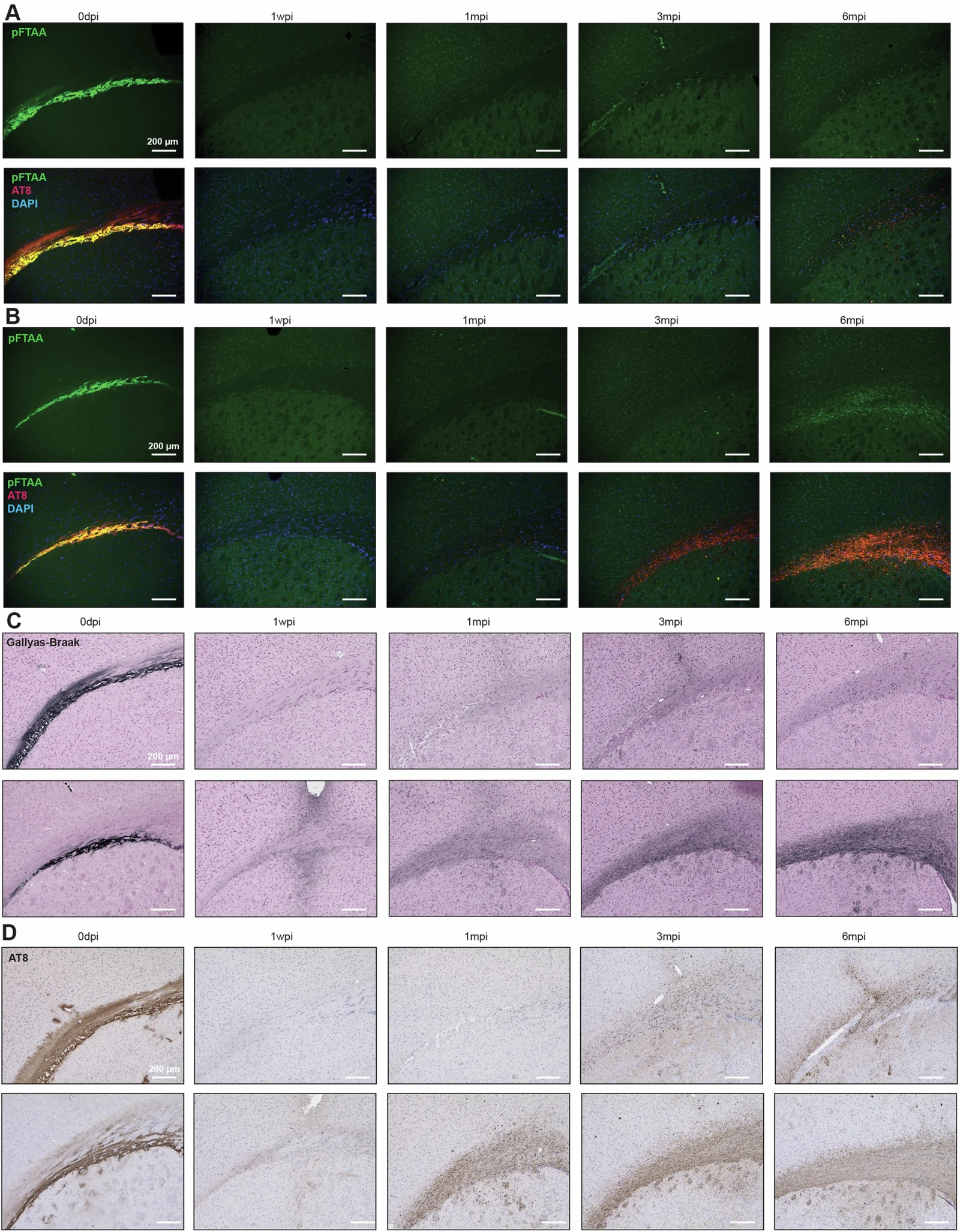
Time course immunofluorescence and immunohistochemistry of AD and CBD seed-injected mice. pFTAA and AT8 immunofluorescence staining of the corpus callosum at the level of the frontal cortex from AD (**A**) and CBD (**B**) seed-injected mice immediately after injection (0 dpi), 1 week post-injection (1 wpi), 1month post-injection (1 mpi), 3 mpi and 6 mpi. (**C**) Gallyas-Braak silver staining of AD (top) and CBD (bottom) seed-injected mice at 0 dpi, 1 wpi, 1 mpi, 3 mpi and 6 mpi. (**D**) AT8 immunohistochemistry staining of AD (top) and CBD (bottom) seed-injected mice. Scale bar, 200 µm.

**Extended Data Fig. 5:**
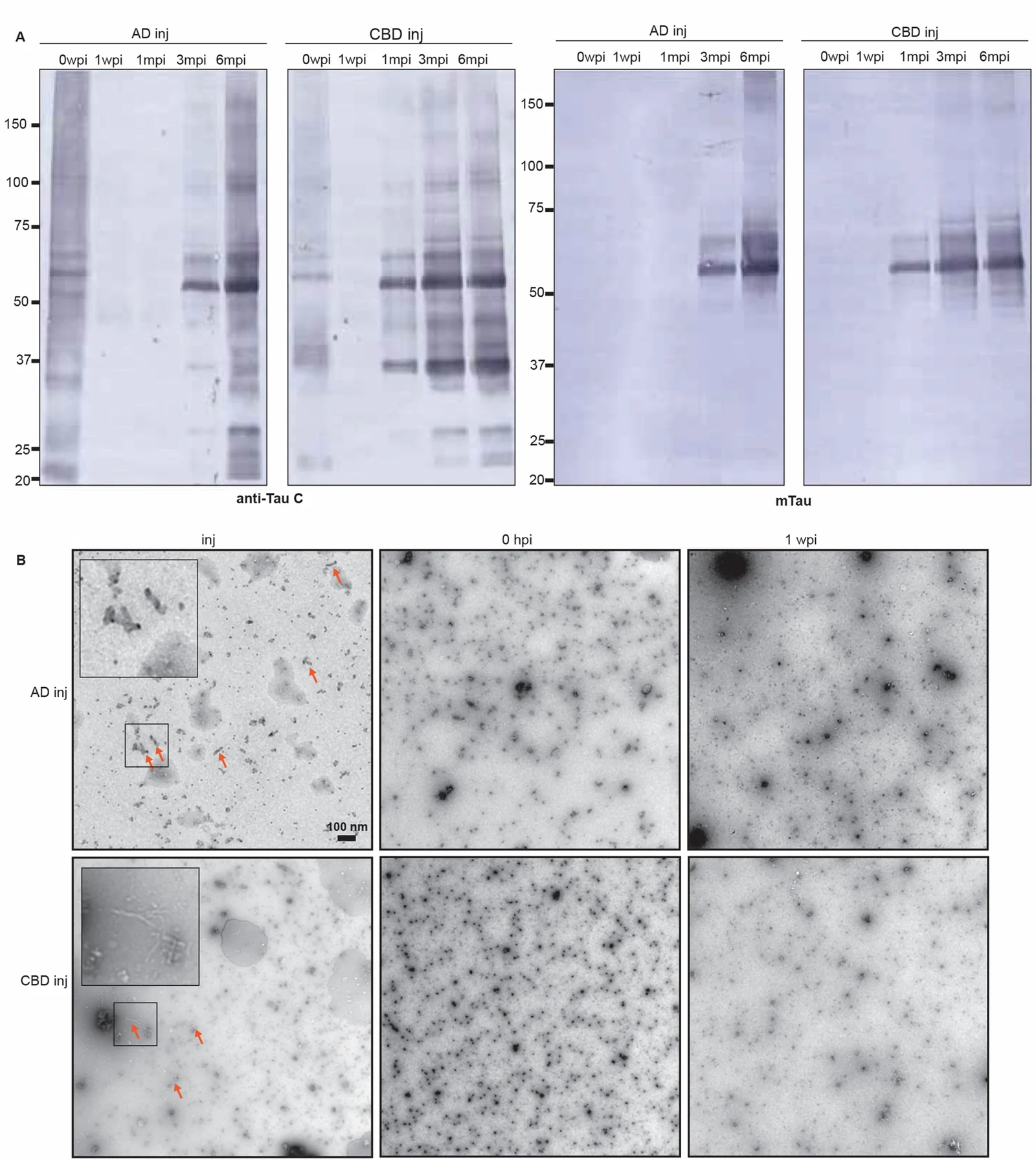
Time course Western blotting and negative stain electron microscopy of AD and CBD seed-injected mice. **A** Western blotting of sarkosyl-insoluble fractions from brain hemispheres of AD and CBD-injected mice at 0, 1 wpi, 1 mpi, 3 mpi and 6 mpi using antibodies anti-Tau C and mTau. **B** Negative stain electron microscopy of the AD and CBD seeds used for injections (inj), immediately after injection (0hpi) and 1 wpi. Arrows point to filaments. Scale bar, 100 nm.

To further eliminate the possibility that the injected human tau filaments remained intact during the seeding experiments, we performed a time-course analysis of the injected seeds using histological staining, electron microscopy and western blotting. The human tau seeds were visible by staining immediately after injection, but they could no longer be detected after 1 week. AT8, pFTAA and Gallyas–Braak silver staining showed tau inclusions in mice injected with CBD seeds at 1 mpi and in mice injected with AD seeds at 3 mpi. In both AD- and CBD-injected mice, tau inclusions had increased in number by 6 mpi and 9 mpi, respectively (Extended Data Fig. 4). Clearance of the injected human seeds and time-dependent increase in mouse tau inclusions was confirmed by negative-stain electron microscopy and western blotting using anti-Tau C, mTau and AT8 antibodies (Extended Data Fig. 5).

## Seeded filaments identical to the seeds

**Fig. 3:**
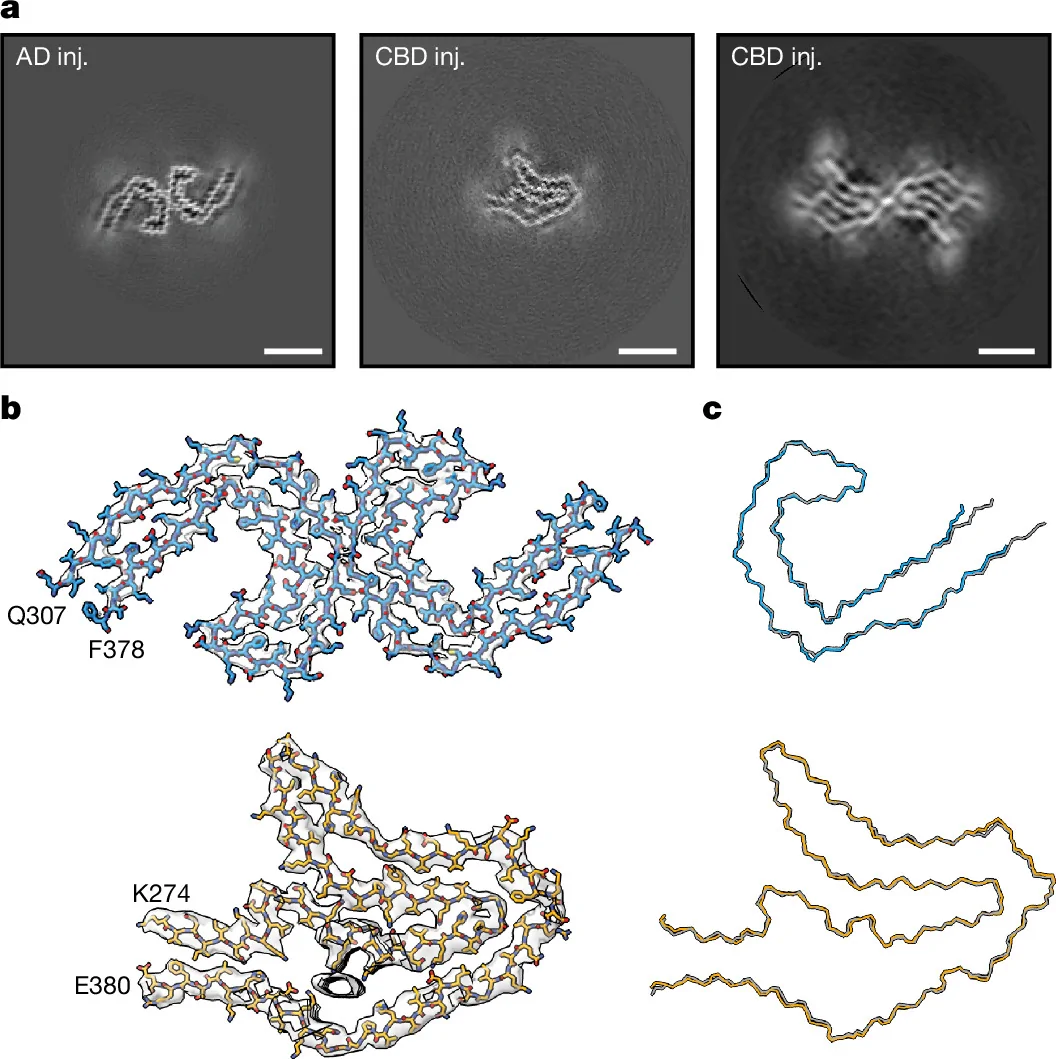
Cryo-EM structures of seeded mouse tau filaments. **a**, Cryo-EM cross-sections of AD- and CBD-seed-injected mouse tau filaments perpendicular to the helical axis, each representing one rung. Scale bars, 5 nm. **b**, The atomic model fitted into the cryo-EM density map (grey transparent) of the AD- (top, blue) and CBD-seed-injected (bottom, orange) mouse tau filaments. **c**, Overlay of the C-alpha backbone of AD- (top, blue) and CBD-seed-injected (bottom, orange) mouse tau filaments overlaid with human ex vivo AD PHFs and CBD type I filaments (grey). The respective AD and CBD structures overlap with a root mean square deviation for all non-hydrogen atoms of 0.906 and 0.576 Å.

**Extended Data Fig. 6:**
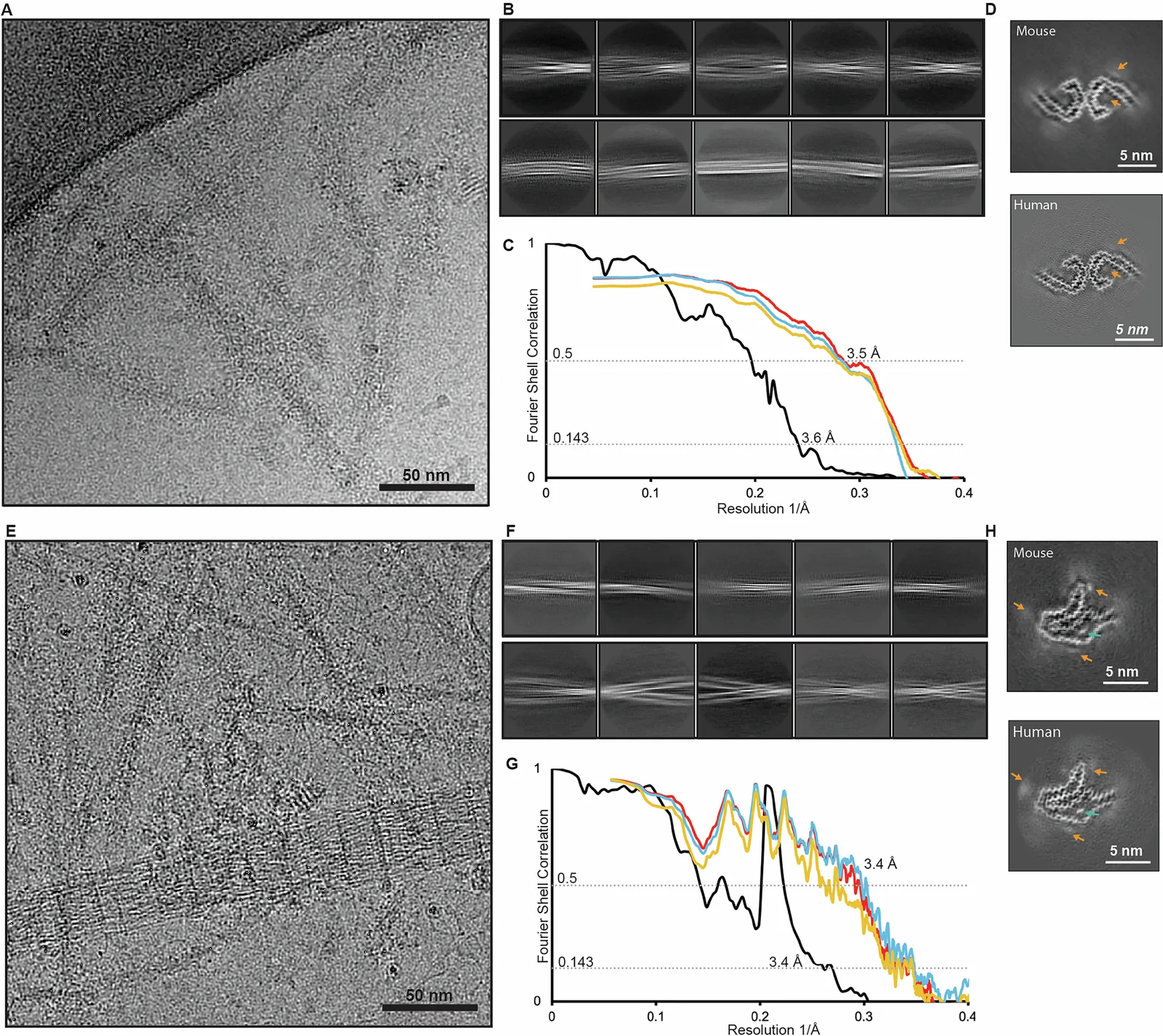
Cryo-EM data processing of seeded mouse tau filaments. **A-D** For AD and **E-H** for CBD seed-injected mice. **A**, **E** Representative cryo-EM micrographs. Scale bar, 50 nm. **B**, **F** Representative 2D class averages; the box sizes are 630 Å. **C**, **G** Fourier shell correlation curves (FSC) between two independently refined half-maps (black); between the atomic model fitted in half-map 1 and half-map1 (blue); between the atomic model fitted in half-map 1 and half-map 2 (yellow) and between the atomic model fitted in the sum of the two half-maps and the sum of the two half-maps (red). **D**, **H** Cross-sections through cryo-EM density maps of seeded mouse tau (top) and human brain-derived tau filaments (bottom). Similar additional densities at the periphery of the ordered cores are marked with arrows.

Cryo-EM analysis of tau filaments extracted from the brains of mice injected with AD or CBD seeds demonstrated that seeded mouse tau filaments adopted the same structures as those of the human seeds (Fig. 3, Extended Data Table 4 and Extended Data Fig. 6). In mice injected with AD seeds, most of the filaments (83%) were paired helical filaments (PHFs), for which we calculated a reconstruction to a resolution of 3.6 Å. This structure was identical (with a root mean square deviation for all non-hydrogen atoms of 0.906 Å) to the Alzheimer fold from AD brains^30,31^. We were unable to obtain a reconstruction for the remaining filaments (17%), which showed two-dimensional (2D) class averages that were reminiscent of single protofilaments or of straight filaments from AD (Extended Data Fig. 6b). Cryo-EM images of mouse brains injected with CBD tau seeds contained two filament types: 74% of the filaments were made of a single protofilament and 26% of the filaments were made of two identical protofilaments. We reconstructed the singlets to a resolution of 3.4 Å. This structure was identical (with a root mean square deviation for all non-hydrogen atoms of 0.576 Å) to human type I CBD filaments^32^. A reconstruction of the doublets, to a resolution of 10 Å, suggested that they may be type II CBD filaments.

## Discussion

We provide evidence for the prion-like transmission of human disease-specific tau strains in the mouse brain. Because the mouseand human tau sequences are identical within the ordered cores of the AD and CBD filament folds, mouse tau assembles into filaments with the same atomic structures as those from human brains. Consistent with previous observations^15,17,21,22^, and supporting the hypothesis that distinct tau folds represent unique strains, injection of human-brain-derived tau seeds led to cell-type-specific lesions in the mouse brain characteristic of AD and CBD.

Alternative splicing of the *MAPT* transcript leads to the expression of six tau isoforms in the adult human brain, with three isoforms comprising three microtubule-binding repeats (3R) and three isoforms comprising four repeats (4R)^33^. Human tauopathies are classified according to the isoform composition of their filaments: a mixture of all six isoforms assembles in 3R + 4R tauopathies such as AD, only 3R tau assembles in 3R tauopathies such as Pick’s disease, and only 4R tau assembles in 4R tauopathies such as CBD. Because adult mice express only 4R tau, the absence of 3R tau represents a species barrier for the transmission of tau seeds with the Pick fold. This barrier does not apply to 3R + 4R and 4R tauopathies. Therefore, tau seeds from other 3R + 4R and 4R tauopathies, such as CTE and progressive supranuclear palsy, may also be transmitted in wild-type mice. Alternatively, mice expressing both 3R and 4R mouse tau^34^ or mice expressing all six human brain tau isoforms in the absence of endogenous mouse tau^22,35,36^ can be used to transmit seeds from all human tauopathies, and these mice exhibit more extensive tau pathology than wild-type mice^24^.

Despite the similarities in how tau and prion strains propagate, important differences exist between the two proteins. Prion diseases are infectious and can be transmitted between individuals, whereas inter-individual transmission of tauopathies has not been reported (although iatrogenic AD has been reported in recipients of cadaveric pituitary-derived growth hormone that contained AD seeds^37^). It remains to be seen whether the peripheral administration of tau seeds can cause neurodegeneration. Prion diseases typically also progress much faster than tauopathies. Thus, mice injected with prion seeds can develop severe disease^4^, whereas mice injected with tau seeds appear healthy, despite the presence of tau inclusions in the brain^14^. It is possible that mice injected with tau seeds do not live long enough to develop symptoms, or that the amount of tau inclusions that form in these mice is not sufficient to lead to clinical symptoms. It therefore remains unknown whether tau seed propagation and downstream toxicity occur in two separate phases, as has been suggested for prions^38^. Mice that are transgenic for human tau with mutations that cause frontotemporal dementias spontaneously form abundant tau filaments and develop severe neurodegeneration^39–42^. Moreover, rhesus macaques injected with tau seeds from the brains of individuals with the 4R tauopathy progressive supranuclear palsy developed motor and behavioural impairments 6 months after seed injection^43^.

Filament assembly can be separated into three stages. The first stage involves seed uptake, which may involve cell surface receptors^13,44,45^ and is probably influenced by the size of tau filaments and their surface properties, including the presence or absence of a fuzzy coat. The second stage consists of the recruitment and templated conversion of monomeric tau within the recipient cell^46^. This step requires compatibility between the seed, the monomeric tau substrate, including its post-translational modifications, and the cell-type environment, which may or may not comprise cofactors that are essential for filament assembly. During this stage, the human tau seeds disappear progressively, indicating that faithful templating and clearing of tau seeds are co-occurring in brain cells. The third and final stage is the release of the newly formed filaments and their uptake by connected cells, resulting in the stereotypical spread of tau pathology through connected brain regions^47^. Different tau folds may influence one or more of these stages, thereby shaping both the efficiency of propagation and the cell-type specificity observed across tauopathies.

Identifying the molecular factors that modulate tau uptake, templated conversion and propagation is essential for understanding how distinct tau strains transmit specific pathologies in a prion-like manner. Future serial passage experiments will provide a complete characterization of disease-specific tau strains. Our observation that the intracerebral injection of wild-type mice with tau seeds from human brains with neurodegenerative diseases leads to the assembly of mouse tau filaments that retain the conformation of the human disease-specific seeds indicates that the mouse is a suitable model for investigating these mechanisms. This explanation will lead to a better understanding of disease-specific tau strain behaviour and provide new diagnostic and therapeutic avenues.

## Methods

### Tau seeds from AD and CBD brains

Sarkosyl-insoluble fractions were prepared from the brains of five individuals with neuropathologically confirmed AD and six individuals with CBD (Extended Data Table 1). The pellets from about 0.5 g brain samples were washed with 2 ml sterile saline, followed by ultracentrifugation as described^48^. The pellets were then sonicated in 120 μl sterile saline using a cup horn sonicator (Sonifier SFX, Branson) at 35% power for 180 s (ref. ^49^). These were the AD and CBD seeds that were used for injection. Informed consent was obtained from the individuals who donated their brains for the study. Human brain tissue was used with permission from the ethical review boards of the relevant institutions and hospitals.

### Intracerebral injection of AD and CBD seeds

Wild-type mice (6–18 weeks old) were used (see Extended Data Tables 2 and 3 for numbers of mice used; no statistical considerations were used to determine sample size, and no randomization was used in the design of this study). They were housed under standard conditions with ad libitum access to food and water. Under isoflurane anaesthesia, C57BL/6J male mice (Japan SLC) were fixed in a stereotaxic frame and injected with 3 μl or 5 μl seeds into the striatum bilaterally (coordinates from bregma: ±0.5 mm anterior, ±2.0 mm lateral and −3.0 mm depth) using a Hamilton syringe. Following injection, the animals were returned to their cages and analysed 9–12 months later. For cryo-EM, two mouse brains injected with either AD or CBD tau seeds were pooled. All experimental protocols were approved by the Animal Care and Use Committee of the Tokyo Metropolitan Institute of Medical Science.

### Immunohistochemistry

Mice were anaesthetized with mixed anaesthetic (0.75 mg kg^−1^ of medetomidine, 4 mg kg^−1^ of midazolam and 5 mg kg^−1^ of butorphanol), perfused with saline, and their brains were collected. For histological analysis, brains were fixed with 10% formalin neutral buffer solution (Wako). Fixed brains were then embedded in paraffin and sectioned at 8 μm. The sections were deparaffinized in xylene and rehydrated through a graded ethanol series. For high-sensitivity detection, sections were autoclaved at 121 °C for 10 min and treated with formic acid for 5 min, followed by incubation in 3% hydrogen peroxide in phosphate-buffered saline for 15 min to inactivate peroxidases. After blocking with 10% calf serum in phosphate-buffered saline with 0.1% Triton X-100, sections were incubated overnight at 4 °C with primary antibodies. They were then incubated with biotinylated secondary antibodies (1:1,000, Vector Laboratories) for 2 h. Immunolabelling was visualized using 3,3′-diaminobenzidine and an ABC staining kit (Vector Laboratories). Sections were counterstained with haematoxylin. Images were taken using a BX53 microscope equipped with a DP80 camera (Olympus). The following primary anti-tau antibodies were used: phosphorylation-dependent (AT8, mouse monoclonal, 1:1,000, Invitrogen; AT100, mouse monoclonal, 1:1,000, Invitrogen), a mouse tau-specific antibody (mTau, rabbit polyclonal, 1:1,000, Cosmo Bio), a conformational antibody (GT-38, mouse monoclonal, 1:1,000, Abcam), and a human tau-specific antibody (HT7, mouse monoclonal, 1:2,000, Invitrogen). For double-label immunofluorescence, deparaffinized sections were incubated overnight at 4 °C with primary antibodies (AT8, mouse monoclonal, 1:1,000, Invitrogen; NeuN, rabbit polyclonal, 1:1,000, Abcam; GFAP, rabbit polyclonal, 1:500, Abcam; Olig2, rabbit monoclonal, 1:500, Abcam). The sections were then washed and incubated with a cocktail of Alexa Fluor 594-conjugated goat anti-mouse IgG, Alexa Fluor 488-conjugated goat anti-rabbit IgG (1:500; Vector Laboratories) and DAPI (1:1,000; DOJINDO) for nuclear counterstaining. Staining with pentameric formyl thiophene acetic acid (pFTAA; Sigma-Aldrich) was performed by incubating the sections with 6 μM pFTAA for 1 h at room temperature following AT8 immunohistochemistry. After further washing, the sections were coverslipped with anti-fade mounting medium (Vectashield, Vector Laboratories) and observed using a BZ-X710 microscope (Keyence).

### Modified Gallyas–Braak silver staining

Deparaffinized sections were oxidized in 0.25% potassium permanganate solution for 15 min. After washing, the sections were bleached in 2% oxalic acid solution. The sections were then immersed for 1 min in silver iodide solution (100 ml distilled water, 4 g sodium hydroxide, 10 g potassium iodide, 1.75 ml 1% silver nitrate). After rinsing with 0.5% acetic acid, the sections were incubated in developer solution for 15–20 min. The reactions were stopped with 0.5% acetic acid, followed by washing and incubation in 1% gold chloride solution for 5 min. After washing, the sections were incubated in a fixer solution for 10 min. Sections were counterstained with 0.1% Kernechtrot. Images were taken using a BX53 microscope equipped with a DP80 camera (Olympus).

### Biochemical and ultrastructural analyses of tau filaments

Mouse brains were frozen on dry ice and stored at −80 °C. Sarkosyl-insoluble pellets were prepared from about 0.5 g tissue, as described in ref. ^50^. The pellets were sonicated in 60 μl saline and aliquots used for immuno-EM. Thirty microlitres of the samples were treated with 30 μl of 2× sample buffer at 100 °C for 3 min, and about 5–10 μl were loaded for immunoblotting. Immunoblotting and immuno-EM were performed using anti-tau antibodies T46 (1:1000), AT8 (1:1,000), mTau (1:2,000), human tau-specific antibody HT7 (1:2,000) and an antibody specific for the C-terminus of tau (anti-Tau-C, rabbit polyclonal, 1:3,000, Cosmo Bio), as described in ref. ^48^. Sarkosyl-insoluble fractions extracted from the injected mice were dropped onto carbon-coated 300-mesh copper grids (Nissin EM) and dried. The grids were stained with mTau or AT8 (1:50). Secondary antibody was conjugated to 5 nm gold particles (Cytodiagnostics, 1:100). A drop of 2% phosphotungstic acid was added to the stained grids, and electron micrographs were recorded with a JEOL JEM-1400 electron microscope.

### Cryo-EM

Three microlitres of sarkosyl-insoluble fractions from wild-type mice that had been injected bilaterally with AD or CBD seeds 9–12 months earlier were applied to glow-discharged (Edwards S150B; Edwards High Vacuum International) holey carbon grids (Quantifoil Au R1.2/1.3, 300 mesh) that were subjected to plunge-freezing in liquid ethane using a Vitrobot Mark IV (Thermo Fisher Scientific) at 100% humidity and 4 °C. Cryo-EM images were acquired on a Titan Krios electron microscope operated at 300 kV, equipped with a Falcon4i direct electron detector (Thermo Fisher Scientific) and a Selectris-X energy filter. Images were recorded at a dose of 40 electrons per Å^2^ using EPU software (Thermo Fisher Scientific).

### Cryo-EM image processing

Datasets were processed in RELION using amyloid helical reconstruction procedures^51^. Movie frames were gain-corrected, aligned and dose-weighted using our own implementation of the motion correction program of RELION^52^. Contrast transfer function (CTF) parameters were estimated using CTFFIND-4.1 (ref. ^53^). Filaments were picked automatically and extracted in boxes of 768 × 768 pixels, which were downscaled to 128 × 128 pixels. Reference-free 2D classification followed by bi-hierarchical classification^54^ was used to separate distinct filament types and select classes suitable for reconstruction. Standard three-dimensional (3D) auto-refinements were performed, including optimization of the helical twist and rise parameters. Bayesian polishing^55^ and CTF refinement^56^ were used to improve the resolution of the reconstructions. Final maps were sharpened using standard postprocessing procedures, and their resolutions were calculated based on the Fourier shell correlation between two independently refined half-maps at a threshold of 0.143 (ref. ^57^) (Extended Data Fig. 6). Atomic models of human-brain-derived tau filaments (PDB-IDs 6HRE^31^ for PHFs and 6TJO^32^ for CBD type I filaments) underwent rigid-body fitting into the maps and were refined using ISOLDE^58^ (Extended Data Table 4).

### Ethics statement

The studies carried out at the Tokyo Metropolitan Institute of Medical Science were approved through the ethical review process at each institution.

### Reporting summary

Further information on research design is available in the Nature Portfolio Reporting Summary linked to this article.

## Online content

Any methods, additional references, Nature Portfolio reporting summaries, source data, extended data, supplementary information, acknowledgements, peer review information; details of author contributions and competing interests; and statements of data and code availability are available at https://doi.org/10.1038/s41586-026-11061-x.

## Supplementary information

Supplementary Information This file contains original uncropped western blot images for Fig. 2a, original uncropped western blot images for Extended Data Fig. 1b and original uncropped western blot images for Extended Data Fig. 5a.

Reporting Summary

Peer Review File

## Figures and Tables

**Extended Data Table 1:**
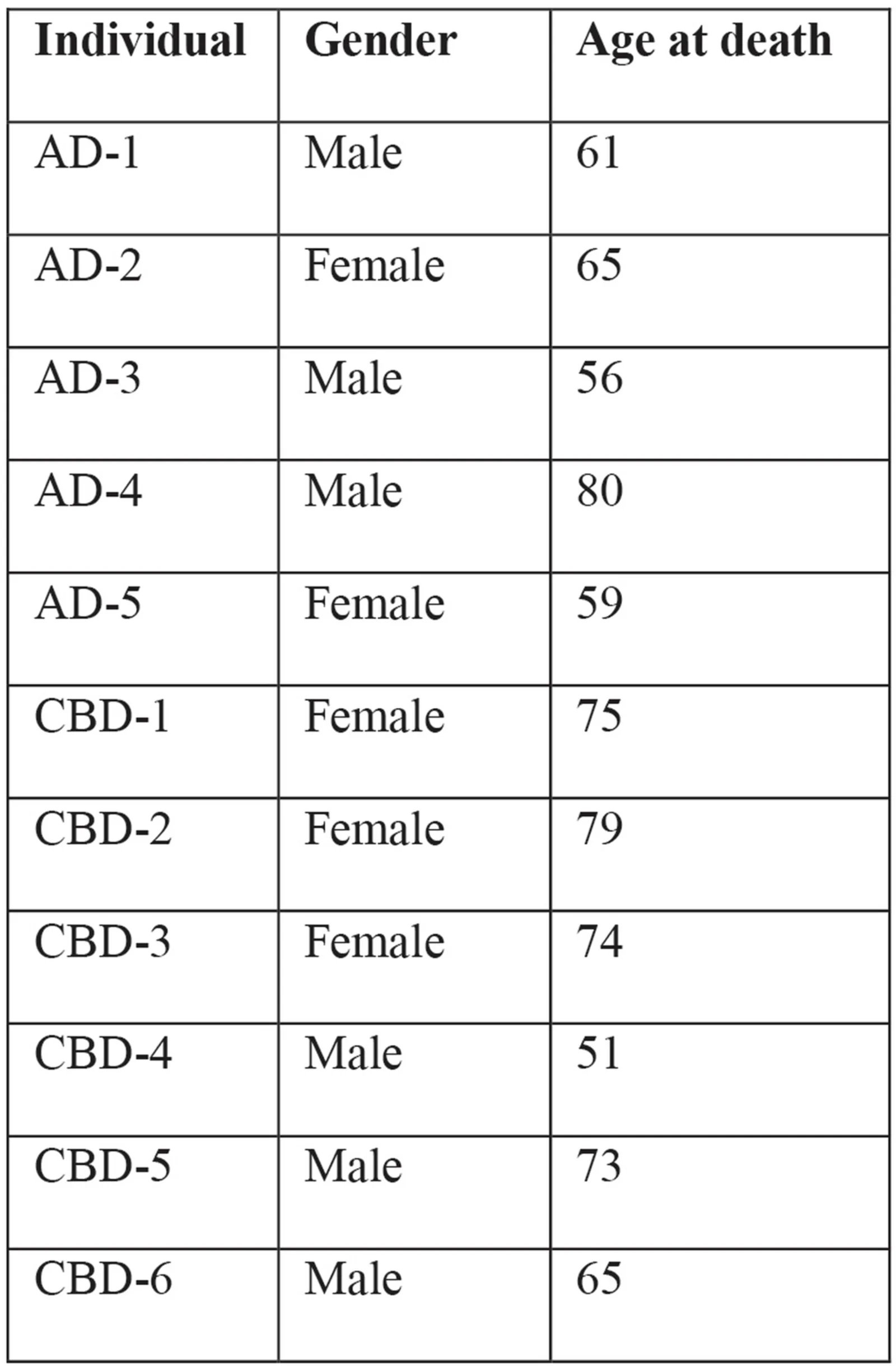
Characteristics of AD and CBD cases used in this study

**Extended Data Table 2:**
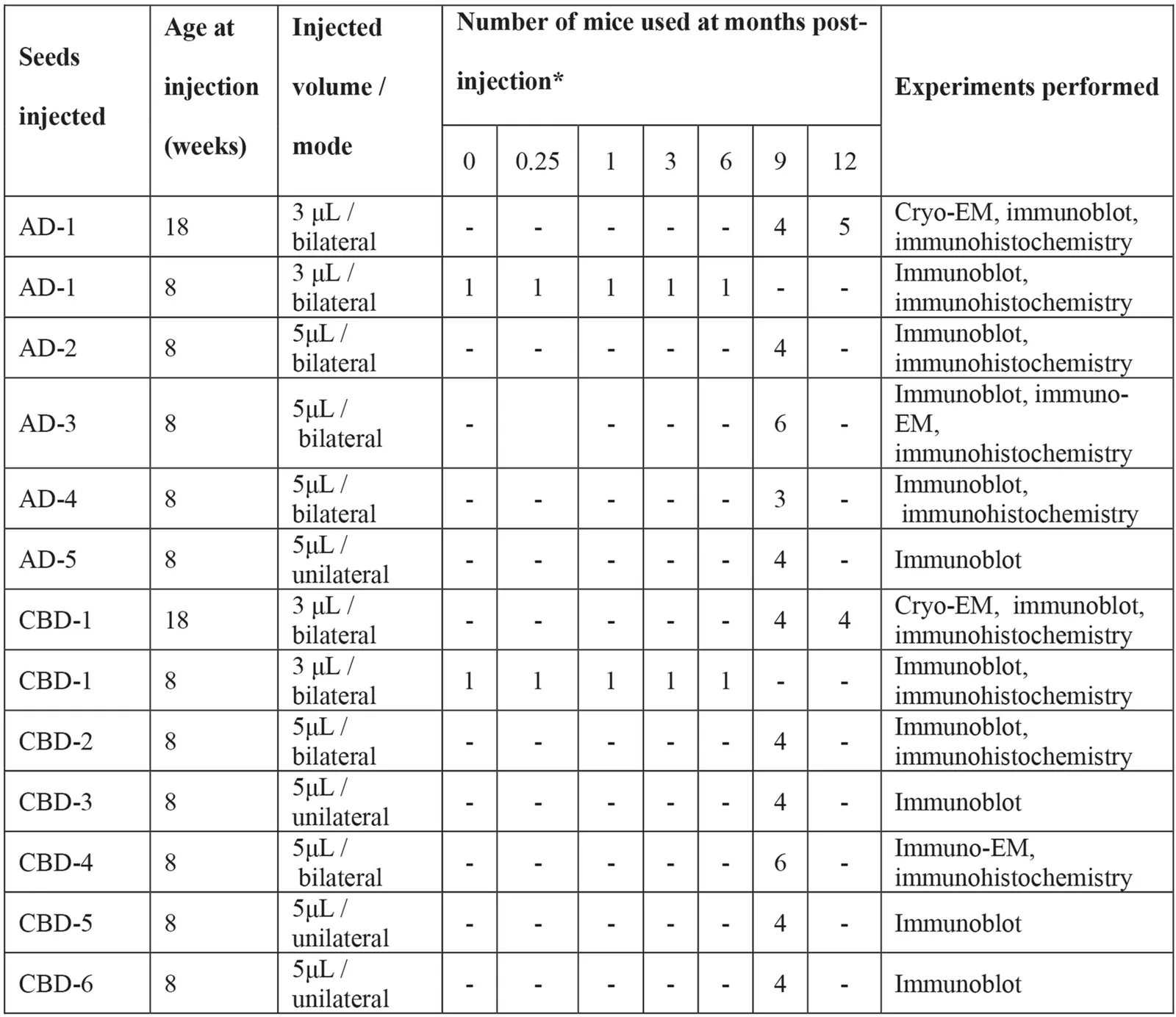
Mice used in this study

Experiments at 0 months were performed on the day of injection; experiments at 0.25 months were performed one week after injection.

**Extended Data Table 3:**
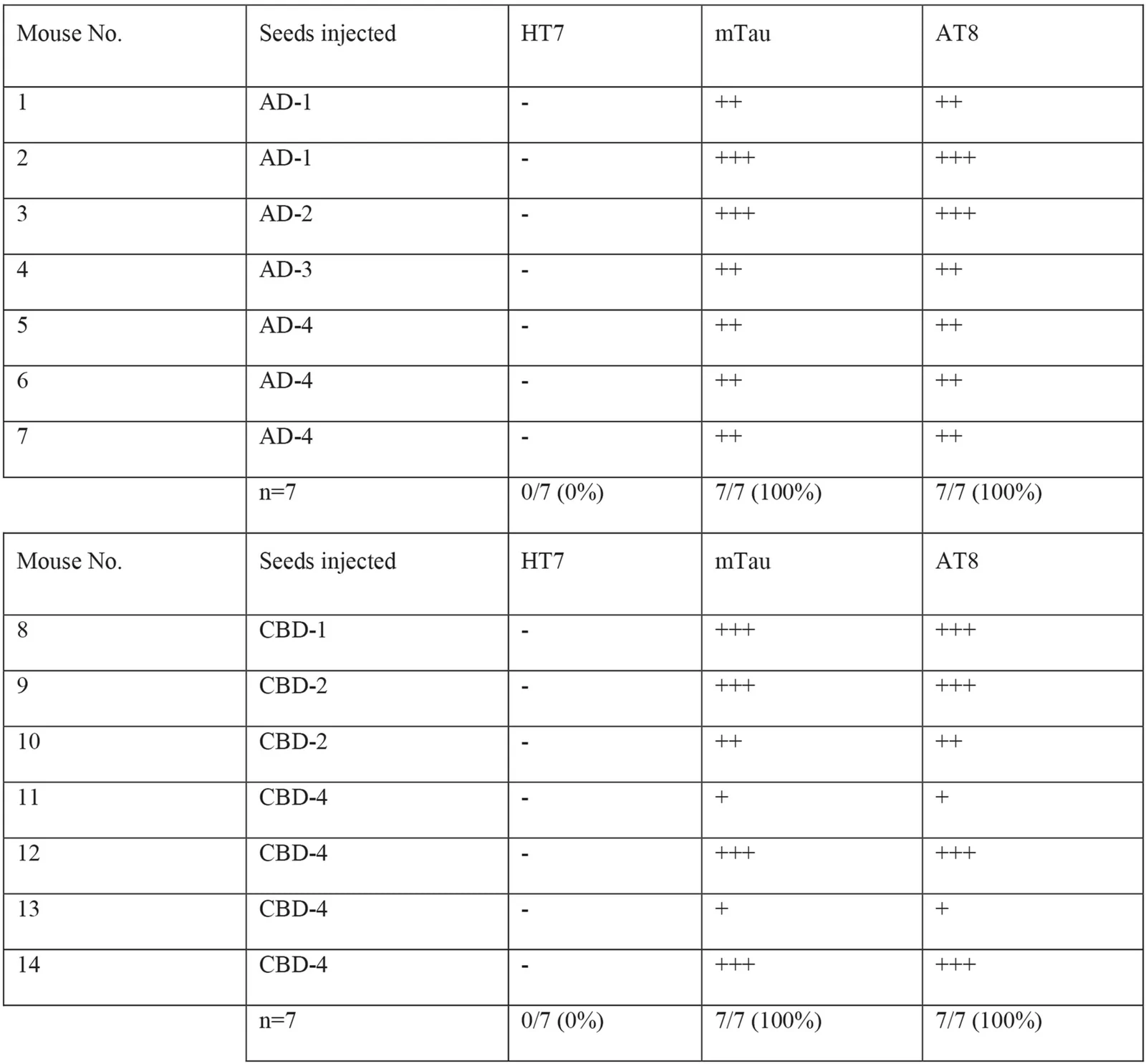
Summary of Tau pathology in mice injected with AD and CBD seeds at 9 mpi

Coronal sections were stained with HT7 and mTau, AT8 antibody at 9 mpi. Grading of tau pathology was performed as follows: -, none; +, slight; ++, moderate; +++, severe. Values show number of immuno-positive mice/total mice, with percentage of immuno-positive mice.

**Extended Data Table 4:**
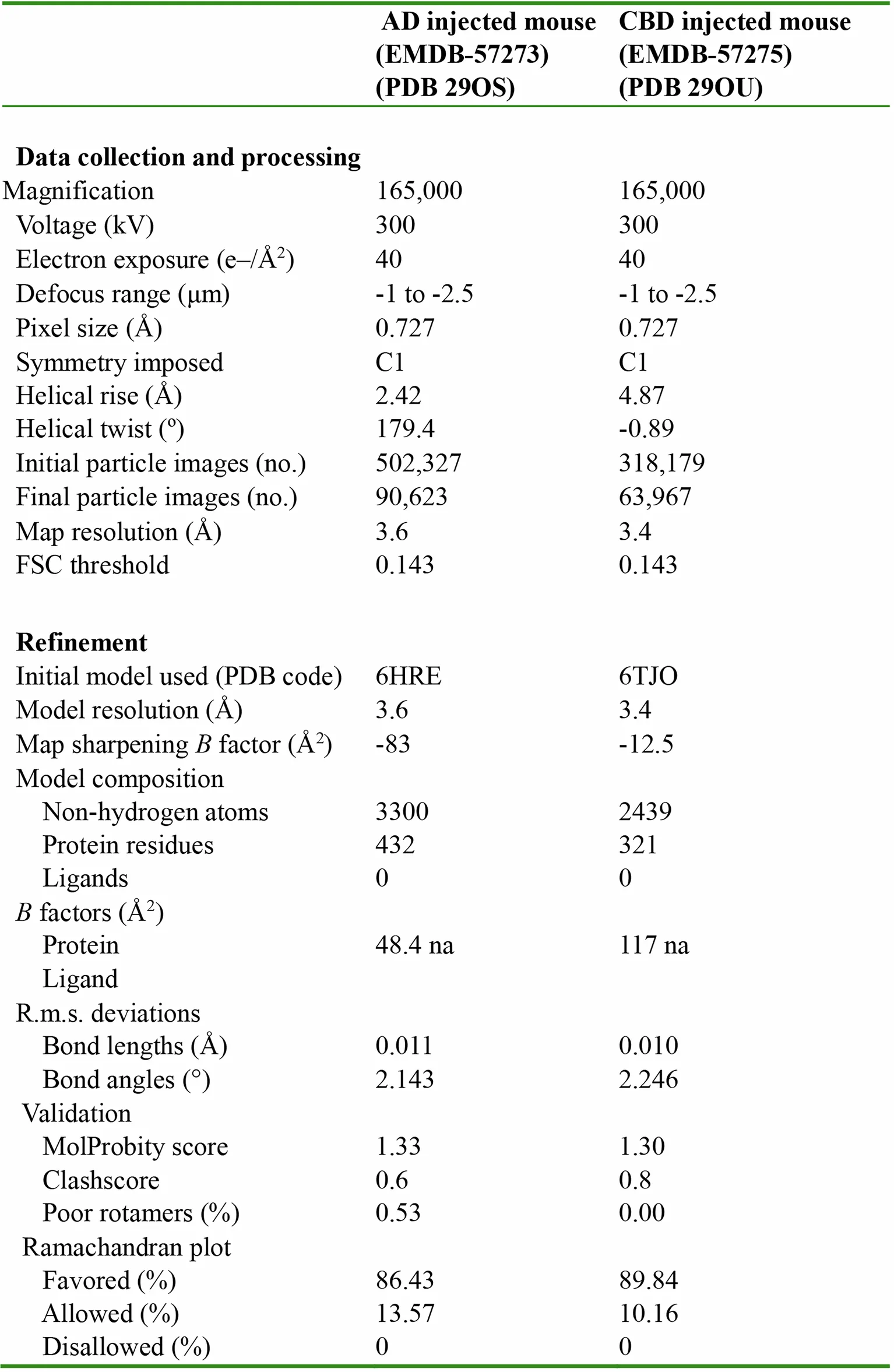
Cryo-EM data collection, refinement and validation statistics

## Data Availability

Atomic coordinates for the mouse tau filaments from the AD- and CBD-seed-injected wild-type mouse brains have been deposited at the Protein Data Bank (accession codes 29OS and 29OU); electron microscopy maps have been deposited at the Electron Microscopy Data Bank (accession codes 57273 and 57275). All other materials are available upon request. Requests for materials should be addressed to M.H., M.G. and S.H.W.S.
