# Supplementary material for: Prion-like transmission of human tau strains in the mouse brain
Source: Nature. 2026 Sep 30;658(8137):972–6. doi: 10.1038/s41586-026-11061-x (PMC13686949; doi:10.1038/s41586-026-11061-x)
Supplement: Supplementary file 1 [file 41586_2026_11061_MOESM1_ESM.pdf]

nature portfolio

https://doi.org/10.1038/s41586-026-11061-x

Supplementary information

Prion-like transmission of human tau strains in the mouse brain

In the format provided by the authors and unedited

1. **Original uncropped western blot images for Figure 2a**

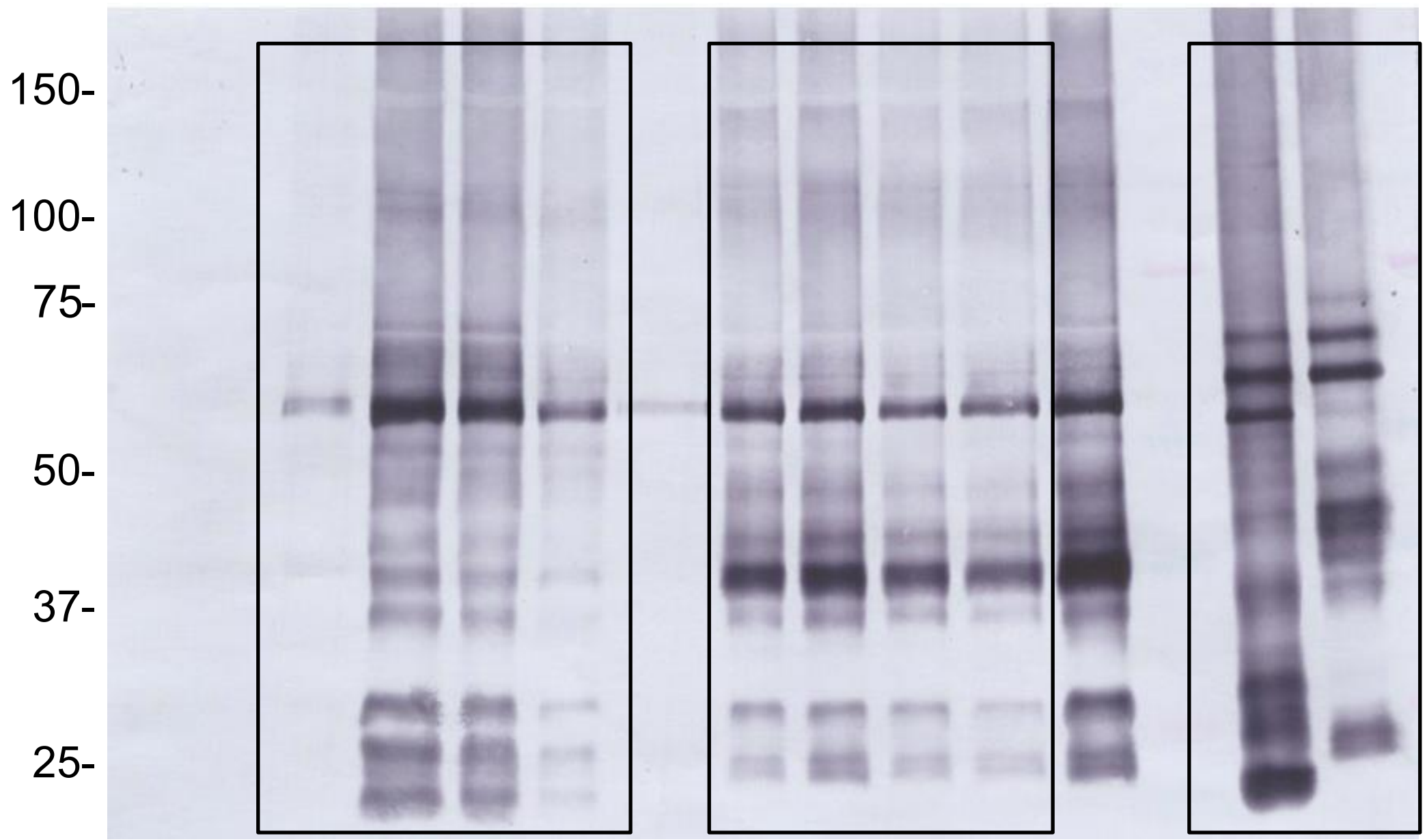

T46

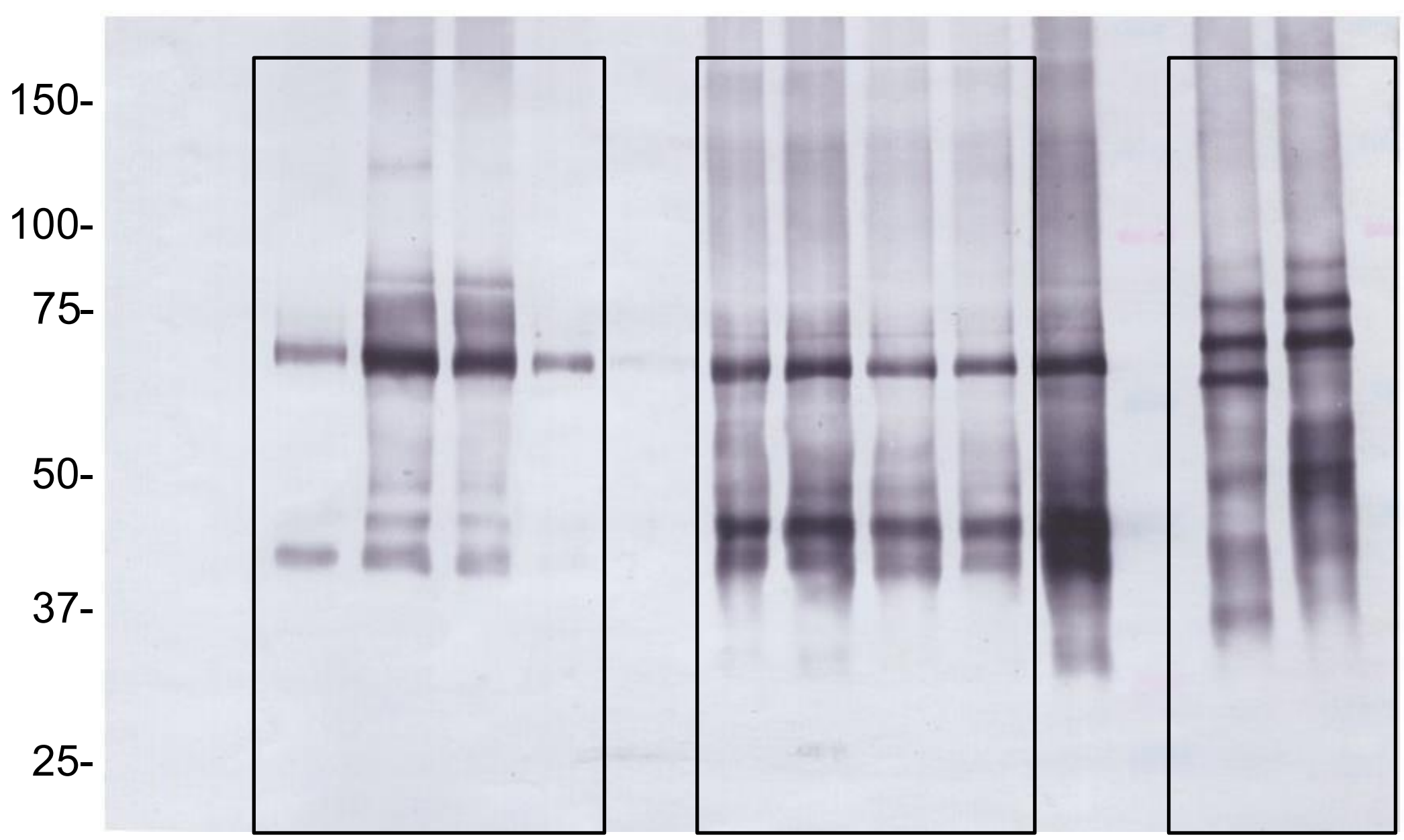

AT8

2. Original uncropped western blot images for Extended Data Figure 1b

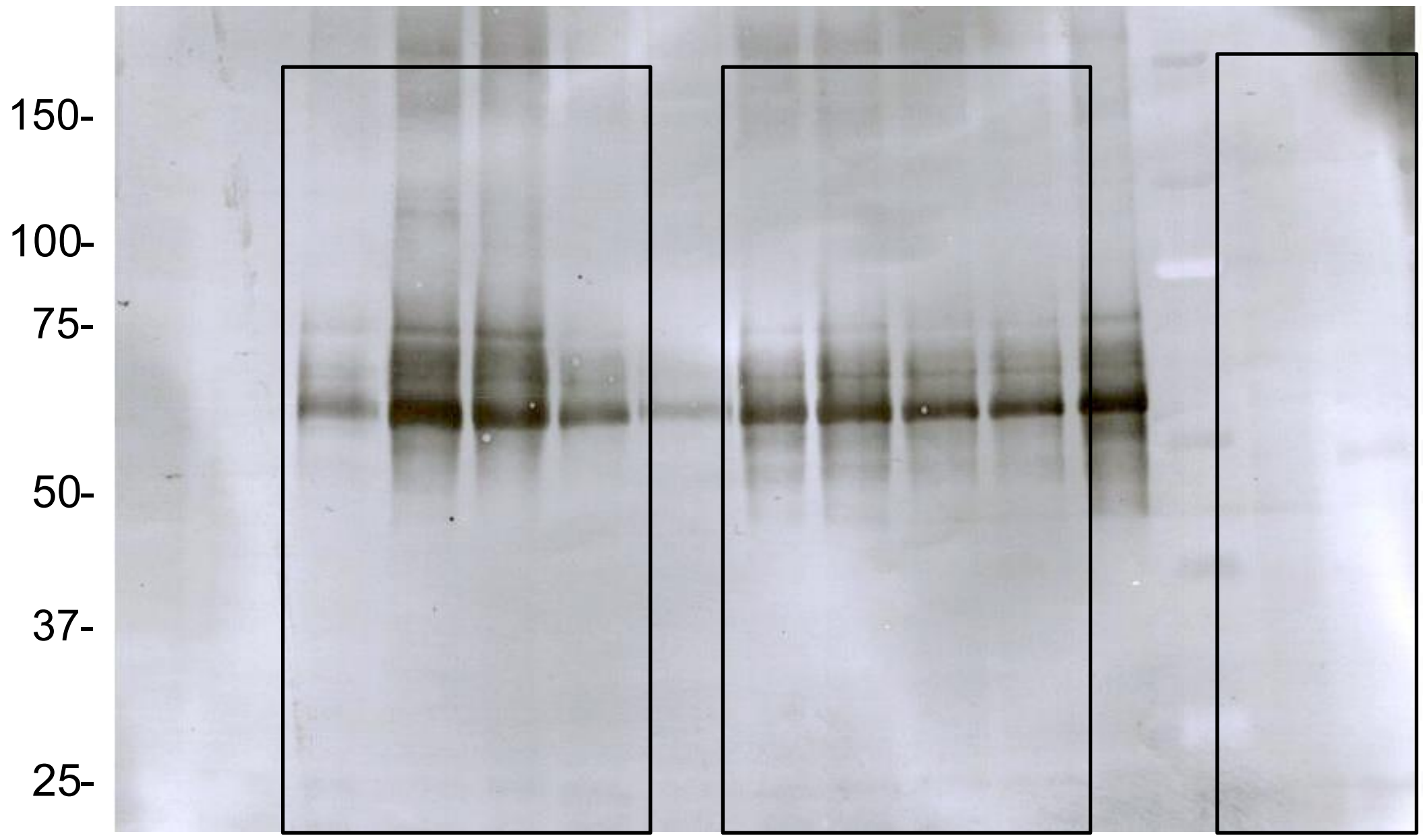

mTau

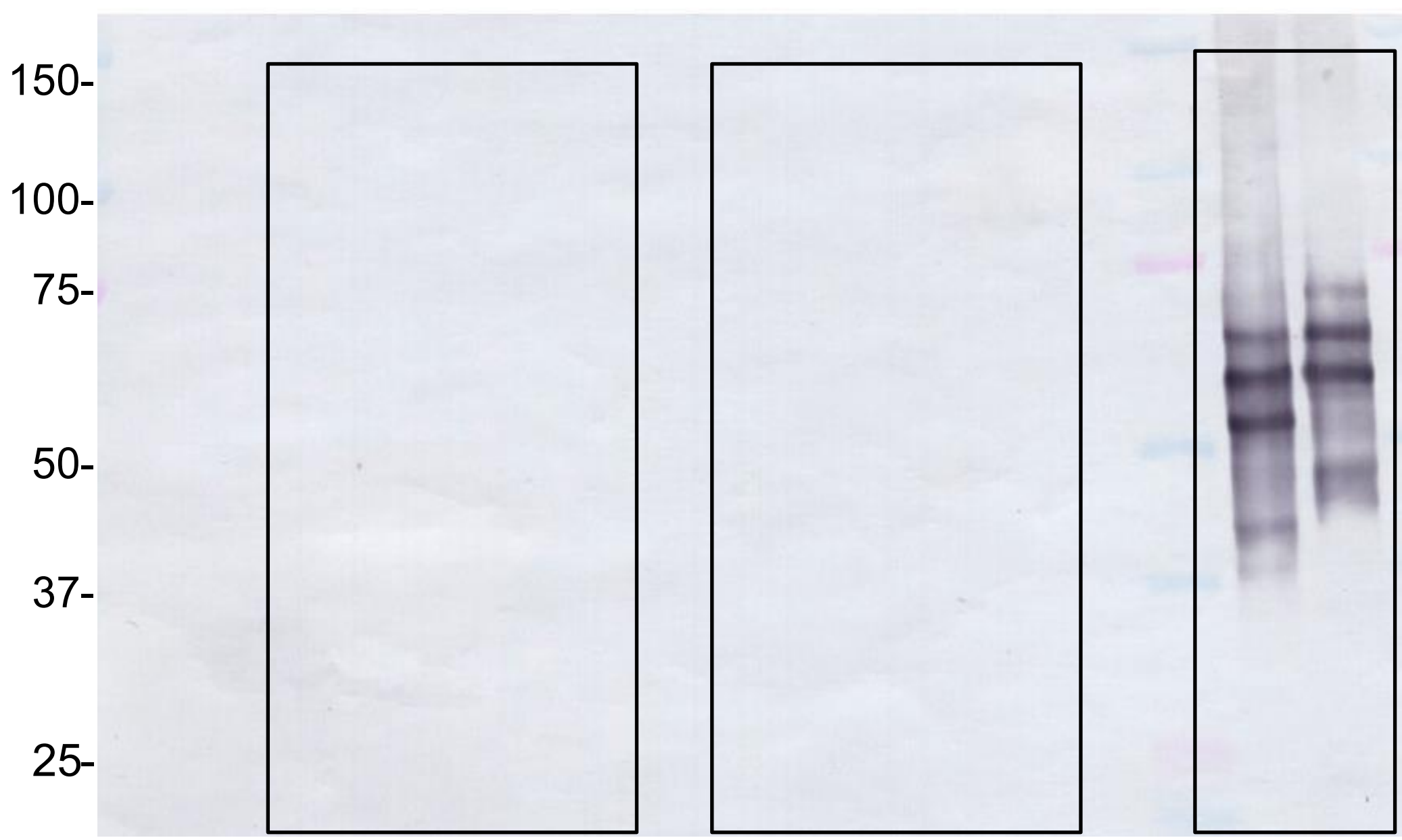

HT7

3. Original uncropped western blot images for Extended Data Figure 5a

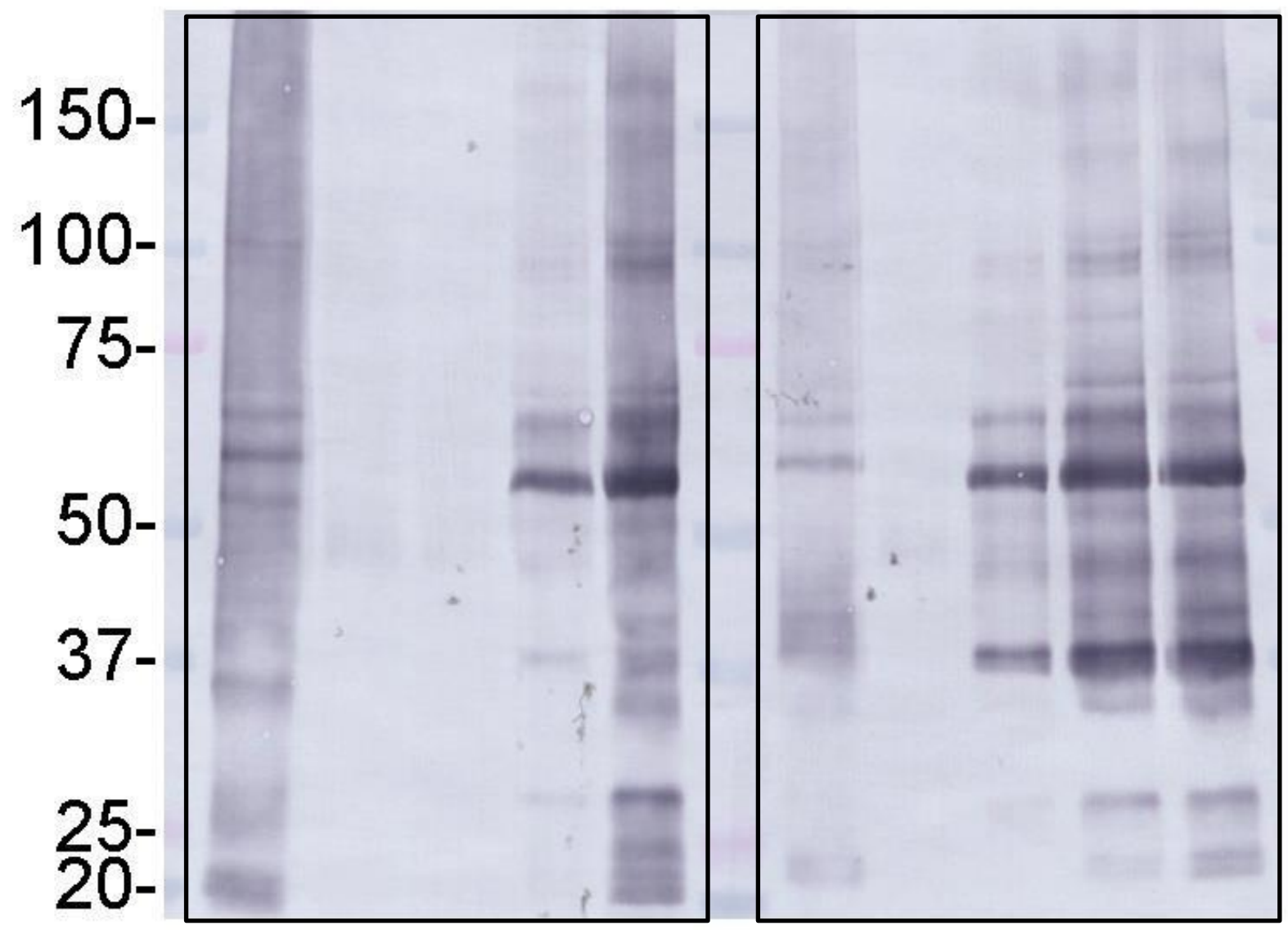

anti-Tau C

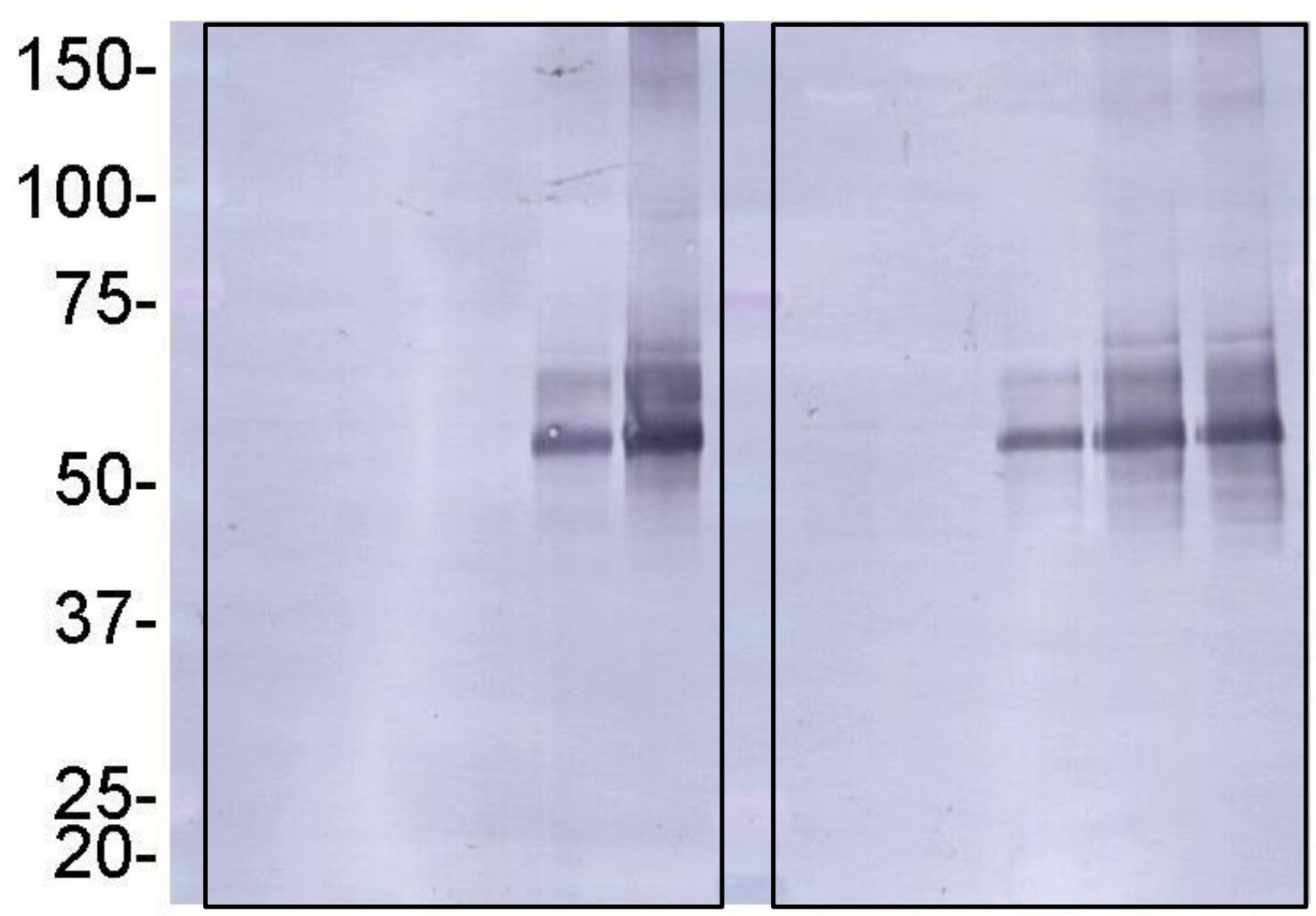

mTau
