# Supplementary material for: Prion-like transmission of human tau strains in the mouse brain
Source: Nature. 2026 Sep 30;658(8137):972–6. doi: 10.1038/s41586-026-11061-x (PMC13686949; doi:10.1038/s41586-026-11061-x)
Supplement: Supplementary file 2 [file 41586_2026_11061_MOESM2_ESM.pdf]

nature portfolio

Corresponding author(s): Masato Hasegawa, Michel Goedert & Sjors Scheres

Last updated by author(s): 08/12/2026

Reporting Summary

Nature Portfolio wishes to improve the reproducibility of the work that we publish. This form provides structure for consistency and transparency in reporting. For further information on Nature Portfolio policies, see our Editorial Policies and the Editorial Policy Checklist.

Statistics

For all statistical analyses, confirm that the following items are present in the figure legend, table legend, main text, or Methods section.

n/a	Confirmed	
☐	☒	The exact sample size (*n*) for each experimental group/condition, given as a discrete number and unit of measurement
☒	☐	A statement on whether measurements were taken from distinct samples or whether the same sample was measured repeatedly
☒	☐	The statistical test(s) used AND whether they are one- or two-sided<br>*Only common tests should be described solely by name; describe more complex techniques in the Methods section.*
☒	☐	A description of all covariates tested
☒	☐	A description of any assumptions or corrections, such as tests of normality and adjustment for multiple comparisons
☒	☐	A full description of the statistical parameters including central tendency (e.g. means) or other basic estimates (e.g. regression coefficient) AND variation (e.g. standard deviation) or associated estimates of uncertainty (e.g. confidence intervals)
☒	☐	For null hypothesis testing, the test statistic (e.g. *F*, *t*, *r*) with confidence intervals, effect sizes, degrees of freedom and *P* value noted<br>*Give P values as exact values whenever suitable.*
☒	☐	For Bayesian analysis, information on the choice of priors and Markov chain Monte Carlo settings
☒	☐	For hierarchical and complex designs, identification of the appropriate level for tests and full reporting of outcomes
☒	☐	Estimates of effect sizes (e.g. Cohen's *d*, Pearson's *r*), indicating how they were calculated

Our web collection on statistics for biologists contains articles on many of the points above.

Software and code

Policy information about availability of computer code

Data collection	EPU (Thermo Fisher)
Data analysis	RELION (open-source: github.com/3dem/relion) version 5.0, CTFFIND-4.1, ChimeraX version 1.10, ISOLDE

For manuscripts utilizing custom algorithms or software that are central to the research but not yet described in published literature, software must be made available to editors and reviewers. We strongly encourage code deposition in a community repository (e.g. GitHub). See the Nature Portfolio guidelines for submitting code & software for further information.

Data

Policy information about availability of data

All manuscripts must include a data availability statement. This statement should provide the following information, where applicable:

- Accession codes, unique identifiers, or web links for publicly available datasets
- A description of any restrictions on data availability
- For clinical datasets or third party data, please ensure that the statement adheres to our policy

Atomic coordinates for the mouse tau filaments from the AD and CBD-seed injected wildtype mouse brains have been deposited at the Protein Data Bank (accession codes 29OS and 29OU electron microscopy maps have been deposited at Electron Microscopy Data Bank (accession codes 57273 and 57275). The structures that were used as an initial models are available in the Protein Data Bank (accession codes 6HRE and 6TJO). All other materials are available upon request

Research involving human participants, their data, or biological material

Policy information about studies with human participants or human data. See also policy information about sex, gender (identity/presentation), and sexual orientation and race, ethnicity and racism.

Reporting on sex and gender	Gender and age of death of individuals with AD or CBD are reported in Extended Data Table 1.
Reporting on race, ethnicity, or other socially relevant groupings	Unknown.
Population characteristics	NA
Recruitment	NA
Ethics oversight	Human brain tissue was used with permission from the ethical review boards of the relevant institutions and hospitals.

Note that full information on the approval of the study protocol must also be provided in the manuscript.

Field-specific reporting

Please select the one below that is the best fit for your research. If you are not sure, read the appropriate sections before making your selection.

☒ Life sciences ☐ Behavioural & social sciences ☐ Ecological, evolutionary & environmental sciences

For a reference copy of the document with all sections, see nature.com/documents/nr-reporting-summary-flat.pdf

Life sciences study design

All studies must disclose on these points even when the disclosure is negative.

Sample size	Numbers of mice used are reported in Extended Data Table 2. The sample size was sufficient for pooling and determining the cryo-EM structure
Data exclusions	Suboptimal cryo-EM particle images were discarded using standard 2D and 3D classification algorithms.
Replication	Seven mice each were injected with AD and CBD 9 mpi for biochemical and cryo-EM analysis and confirmed the prion-like spreading of the mouse tau with several measures. The replication choice was a measure of feasibility and confirmation of the finding
Randomization	NA as the study required biochemical and structural analysis
Blinding	NA as the study required biochemical and structural analysis

Reporting for specific materials, systems and methods

We require information from authors about some types of materials, experimental systems and methods used in many studies. Here, indicate whether each material, system or method listed is relevant to your study. If you are not sure if a list item applies to your research, read the appropriate section before selecting a response.

Materials & experimental systems

n/a	Involved in the study	
☐	☒	Antibodies
☒	☐	Eukaryotic cell lines
☒	☐	Palaeontology and archaeology
☐	☒	Animals and other organisms
☒	☐	Clinical data
☒	☐	Dual use research of concern
☒	☐	Plants

Methods

n/a	Involved in the study	
☒	☐	ChIP-seq
☒	☐	Flow cytometry
☒	☐	MRI-based neuroimaging

Antibodies

Antibodies used	The following primary anti-tau antibodies were used: phosphorylation-dependent (AT8, mouse monoclonal, 1:1000; Invitrogen; AT100, mouse monoclonal, 1:1000; Invitrogen), a mouse tau-specific antibody (mTau, rabbit polyclonal, 1:1000; Cosmo Bio), a conformational antibody (GT-38, mouse monoclonal, 1:1000; Abcam), a human tau-specific antibody (HT7, mouse monoclonal,

	1:2000; Invitrogen) and an antibody specific for the C-terminus of tau (Anti Tau-C, rabbit polyclonal, 1:3000; Cosmo Bio). Immunoblotting and immuno-EM were performed using anti-tau anti-bodies T46 (1:1000), AT8 (1:1000), mTau (1:2000), and human tau-specific antibody HT7 (1:2000).
Validation	A T8 (https://www.thermofisher.com/antibody/product/Phospho-Tau-Ser202-Thr205-Antibody-clone-AT8-Monoclonal/MN1020),<br>AT100 (https://www.thermofisher.com/antibody/product/Phospho-Tau-Thr212-Ser214-Antibody-clone-AT100-Monoclonal/MN1060)<br>mTau (Masuda-Suzukake et al., Brain Comm., 2020),<br>GT-38 (https://www.abcam.eo.jp/products/primary-antibodies/tau-a lzh e i m ers-d i sea se-a nti body-gt -38-co nformati on-specific-ab 2 46808),<br>HT7 (https ://www. thermofisher.com/anti body/ product/Tau-Anti body-clon e-HT7-M o noclonal/MN1000),<br>Tau-C(https://search.cosmobio.co.jp/cosmo_search_p/search_gate2/docs/CAC_j CACTI PT AU P04. 230705. pdf),<br>01 ig2 (https://www.abcam.com/en-us/products/primary-antibodies/olig2-antibody-epr2673-oligodendrocyte-marker-ab109186),<br>T46(https://www.thermofisher.com/antibody/product/Tau-Antibody-clone-T46-Monoclonal/13-6400).

Animals and other research organisms

Policy information about studies involving animals; ARRIVE guidelines recommended for reporting animal research, and Sex and Gender in Research

Laboratory animals	C57BL/6J male mice (Japan SLC, Inc.)were used in this study.
Wild animals	No wild animals were used
Reporting on sex	Male animals were used
Field-collected samples	No field samples were collected
Ethics oversight	All experimental protocols were approved by the Animal Care and Use Committee of the Tokyo Metropolitan Institute of Medical Science.

Note that full information on the approval of the study protocol must also be provided in the manuscript.

Plants

Seed stocks	NA
Novel plant genotypes	NA
Authentication	NA
