# Supplementary material for: Prion-like transmission of human tau strains in the mouse brain
Source: Nature. 2026 Sep 30;658(8137):972–6. doi: 10.1038/s41586-026-11061-x (PMC13686949; doi:10.1038/s41586-026-11061-x)
Supplement: Supplementary file 3 [file 41586_2026_11061_MOESM3_ESM.pdf]

nature portfolio

Peer Review File

Prion-like transmission of human tau strains in the mouse brain

Corresponding Author: Dr Sjors Scheres, Masato Hasegawa and Michel Goedert

Any redactions in this file are there to maintain patient confidentiality, the confidentiality of unpublished data, or to remove third-party material.

This file contains all reviewer reports in order by version, followed by all author rebuttals in order by version.

Version 0:

Reviewer comments:

Referee #1

(Remarks to the Author)
It has been proposed for some years that tau assemblies, seen in Alzheimer's disease and a range of tauopathies, may act like prions and there is an extensive literature showing that tau assemblies can seed and spread in animal models following experimental inoculation with tissue extracts from human patients. In the current manuscript, they focus on the strain phenomenon which is central to prion pathobiology where structurally distinct prion protein fibrillar assemblies relate to distinct clinicopathological phenotypes in inoculated animals which are stable on serial passage.

They intracerebrally inoculated groups of wild type mice with brain extracts from patients with either AD or CBD, distinct neurodegenerative diseases both associated with tauopathy. They show distinct patterns of pathology in these mice (and different rates of its development) which associate with distinct tau amyloid structures indistinguishable for those in the human brains. They argue that tau filaments propagate by templated seeding akin to prion propagation and that the mouse provides a good model to study the mechanisms by which distinct tau folds produce specific patterns of neuropathology.

This is an important and insightful manuscript from distinguished authors which provides significant further evidence for the importance of prion-like mechanisms in the common neurodegenerative diseases. However, I do have a few queries and suggestions for further improving the manuscript:

1. ED Table 2 shows the experimental design. It is not clear however whether the time course study has 5 mice at each time point or 5 mice in total. There are no further details of these experiments in the methods. n = 1 for the early time points would raise concerns about statistical validity so I am assuming it is n = 5. The authors need to clarify and amend ED Table 2 to make group sizes clear.

2. There is some lack of clarity regarding the investigations in mice, specifically what proportion of inoculated mice showed what pathology. A conventional transmission table with a column for pathological findings would be very helpful.

3. The cryo-EM data look convincing (as would be expected from these authors), and I assume the preliminary PDB validation reports that were submitted will be updated with the official PDB validation reports with the revised manuscript.

4. If tau strains are transmissible to conventional mice, then we should assume they are probably transmissible to humans if similarly inoculated. The circumstances for iatrogenic or laboratory transmission to humans will hopefully be very limited but nonetheless I think the authors should mention this concern in their discussion, not least with respect to laboratory workers handing highly purified seeds prepared for cryo-EM, presumably of high titre. Iatrogenic transmission of AD has been reported (Banerjee et al Nature Medicine 2024) and this could also be cited in this regard.

5. The authors argue that tau seed structure influences both cellular targeting and pathological morphology in the inoculated mice and indeed that they have demonstrated that wild type mice are a suitable model to study the molecular mechanisms by which the distinct tau filament structures drive disease-specific pathology. This may be the case but I would advise some caution here given what we understand about prion pathogenesis in mice. In scrapie prion infected mice two mechanistically

distinct phases are described. Prion propagation rises exponentially early in the incubation period and prion seed titre reaches a plateau well before neuropathology develops (see Sandberg et al Nature 2011 and Nat Comms 2014). However, as these authors note and are seeking to reproduce with tau seeds, distinct prions strains are associated with distinct patterns of neuropathology. This suggests that the neuropathology actually relates to the secondary development of neurotoxic species with selective vulnerability to neurotoxic processes rather than the distinct seeds selectively propagating and targeting different cell types or brain regions. This is beyond the scope of the current manuscript but would be worth mentioning in the discussion.
6. Finally, I should note that conventionally, characterisation of prion strains involves serial passage in a host species, not simply the primary passage described here. Clearly such serial passage is very time consuming and I would not ask the authors to do this with respect to the current manuscript but this should be noted as a limitation in the discussion.

Referee #2

(Remarks to the Author)
The authors report that the intracerebral injection of tau fibrils into wild-type mice induces tau aggregates that adopt the same structure as the injected fibrils. Specifically, mice injected with AD-derived Tau seeds develop pathology predominantly confined to neuronal cell bodies and neurites. In contrast, injection of CBD-derived Tau results in Tau accumulation in both neurons and glial cells, including the formation of coiled bodies and plaque-like astrocytic inclusions.

The manuscript is clearly written, logically organised and easy to follow. The experiments are carefully executed and the conclusions are well supported by the data.

However, the majority of the data (Figures 1–3) reiterates findings that have been reported previously (e.g. reference PMID: 29054878, and others cited in the manuscript), particularly the concept that exogenous tau fibrils act as seeds that template the propagation of their specific conformations in vivo. As such, the central premise of templated Tau propagation is not novel.

The main and only advance of the present study lies in the cryo-EM characterisation of the induced (templated) tau fibrils formed (Figure 4). Demonstrating that these induced (mouse) fibrils faithfully recapitulate the structure of the injected (human) seeds is an important technical and conceptual contribution as it strengthens the validity of mouse models for studying strain-specific human tau propagation.

Nevertheless, this advance may be of greater interest to a more specialised audience. It is less clear whether the findings reach the level of conceptual novelty and general interest required for publication in a widely read journal such as Nature.

Minor comment:
The authors rely primarily on Olig2 staining, which is not sufficient to fully support their claims regarding cell-type specificity.

Referee #3

(Remarks to the Author)
Lövestam and coworkers set out to fundamentally probe the link between the structural fidelity of tau fibril templating in vivo and the pathophysiological consequences of tau aggregates being introduced into mice as a prion strain. The authors consider the tau strain to be an aggregate with a distinct fibril structure. That structure is known at the outset of the study and is specifically identified as that of tau fibrils extracted from the frontal cortex of humans afflicted with AD or CBD. In comparing the effect of injecting either strain into a mouse brain, the authors further delineate potential differences in strain transmission on the basis of fibril structure.
These experiments are in line with recently published work describing the propagation of alpha synuclein fibril seeds in mice, where in vivo templating that largely retained fibril structure was also demonstrated. That type of behavior for alpha synuclein 'strains' was to some extent anticipated by the experiments of Prusiner and co workers, and other groups using transgenic mice to potentiate seeding and limit the species barrier. As the authors well document, many experiments have shown analogous results for tau with transgenic or wild type mice.
Advancing past those studies, the current work clearly establishes the faithful templating of endogenous tau by tau fibril isolates derived from patients and injected into the brains of mice. In that regard, the study is noteworthy and stands out as a resource for the understanding the behavior of tau and other amyloid proteins as prions.

While the work is sufficiently detailed and rigorous to support its conclusions, it brings the following thoughts and questions to mind, which are worth the authors' consideration:

A question is stirred here regarding the protein-only nature of prions. That elegant hypothesis dictates that a protein aggregate acting as a template or seed is sufficient to engender disease by itself. The experiments at hand approximate such an aggregate with fibril seeds extracted from diseased patient brains, but cannot fully account for the role of associated proteins, co-factors or other molecules present in the same fibril fractions. Unlike other studies where recombinant fibrils are injected into mouse brains to cause disease, the presence of unknown agents coexisting with the injected tau seeds introduces a degree of uncertainty into the precise definition of these strains. It may be further complicated by the role played by other tau aggregates (oligomers, minor polymorphs, etc) that may also be present and influence seeding. Are truly pure, monomorphic recombinant fibrils that adopt the same fold sufficient to cause the same strain pathophysiology? Separately, proteomics and/or metabolomics analyses of the two injected strains could be informative in clarifying this point.

The finding that AD and CBD seeds induce distinct patterns of pathology in the mouse brain is noteworthy and perhaps expected. However, the true extent of those differences is difficult to assess. For example, it is not clear from the histological evidence provided whether other aggregated tau species may be present but undetected by the antibodies used.

With regard to GT-38 binding, whose binding site is cleverly suggested to reside in tau R2: The authors note that "the epitope in R2 is accessible for GT-38 binding in the filaments from mouse brains injected with AD tau seeds, but that the same epitope is not accessible in the filaments from mice injected with CBD tau seeds, because it forms part of the ordered filament core." That assumption may be true in the case of GT-38 binding to CBD tau fibrils, but may not generally hold for other tau antibodies, since they could potentially bind motifs in ordered fibril cores. In short, a clearer picture of the GT-38 epitope is be needed to fully support the claims.

The authors claim that their ~10 Å structure of fibrils isolated form mice injected with the CBD strain suggested these were "identical to type II CBD filaments." However, this is difficult to confirm based no the relatively low resolution data being considered.

The lag period described by the authors between the disappearance of exogenous seeds and the detection of nascent tau inclusions is intriguing, particularly given the differences observed in its duration in mice seeded with AD or CBD strains. How do the authors rationalize this discrepancy? Perhaps these two trains face different species barriers?

The evidence for a potential absence of a species barrier in the propagation of human tau strains within wild type mice is particularly noteworthy. As noted by the authors, this may well be rationalized by the similarity in human and mouse tau sequences present within the tau fibril cores in question. However, the impact of sequence differences outside the cores cannot be dismissed and could be the reason behind the lag period observed in nascent tau aggregate formation, particularly for the AD tau strain. It is also noteworthy that the reconstructions of mouse tau fibrils generally lack some of the extraneous, often unmodeled densities observed for patient derived tau fibrils. A comparison of these features could be informative and brings questions about whether the differences are due to the challenges of templating, a species barrier in their retention, or something else entirely?

Since the authors discuss differences in disease onset after challenge with various prion strains, it would be relevant to consider serial passaging of strain isolates in wild type mice, which may further inform on the impact of a potential species barrier and on strain adaptation to a mouse background.

Minor notes:

Figure 4B could benefit from further annotation, e.g. some residue numbering.

Figure 4C may be more informative if the reference chain was colored by RMSD, more easily highlighting the regions of disagreement.

The species being noted in Extended data figure 3 would be more clear if insets were included with higher magnification views.

Version 1:

Reviewer comments:

Referee #1

(Remarks to the Author)
The revised manuscript is significantly improved.

The authors now acknowledge the n=1 limitation of their mouse time course study in ED Table 2 and in the text.

A new transmission table (ED Table 3) is now provided to describe pathology findings in the mice which is helpful.

Regarding the potential for transmission of tau pathology to humans, additional text and a citation has been added regarding the recent recognition of iatrogenic AD. However, their original statement that “prion diseases are infectious and can be transmitted between individuals, whereas inter-individual transmission of tauopathies has not been reported” remains unchanged. Human prion diseases are not transmitted between individuals in normal circumstances, transmission of CJD requires inoculation with human prions during medical or surgical procedures. These iatrogenic CJD cases are very rare and account for only around 1% of CJD. The argument of this paper is that there are strains of tau amyloid that can act as prions and induce strain-specific neuropathology in inoculated mice. If correct, surely it must follow that such human tau strains would also pose a risk to humans, who after all express homologous tau proteins, if there were similarly inoculated. The concern is not of inter-individual transmission as such, but that laboratory workers intentionally purifying tau amyloid seeds for structural or other studies could be at risk from accidental inoculation. I think a clearer but measured statement of potential risk, which logically follows from their own data, is warranted.

The authors have added relevant text in response to my comments 5 and 6.

A further point to mention is regarding IHC panels. Brain sections shown in ED Figure 1 panel A , AD inj 6mpi and ED Figure 4 panel D, AT8 3mpi look as if they could be sections from the same brain and it may be worth double checking in case there was an inadvertent mix up.

Referee #2

(Remarks to the Author)

Referee #3

(Remarks to the Author)
The authors have appropriately addressed the review comments and present an improved manuscript suitable for publication.

My only two remaining comments are:

1. A suggestion to the authors to be quantitative in their comparisons of structures by listing their very low alpha carbon RMSD values, e.g. when stating: "This structure was identical to the
Alzheimer fold from AD brains".

2. A suggestion that the authors consider the process of tau fibril clearance in their discussion paragraph on filament assembly. Their results show that "The human tau seeds were visible by staining immediately after injection, but they could no longer be detected after one week.", suggesting that templating and clearance of fibrils co-occur in cells?

Open Access This Peer Review File is licensed under a Creative Commons Attribution 4.0 International License, which permits use, sharing, adaptation, distribution and reproduction in any medium or format, as long as you give appropriate credit to the original author(s) and the source, provide a link to the Creative Commons license, and indicate if changes were made.
In cases where reviewers are anonymous, credit should be given to 'Anonymous Referee' and the source.
The images or other third party material in this Peer Review File are included in the article’s Creative Commons license, unless indicated otherwise in a credit line to the material. If material is not included in the article’s Creative Commons license and your intended use is not permitted by statutory regulation or exceeds the permitted use, you will need to obtain permission directly from the copyright holder.
To view a copy of this license, visit https://creativecommons.org/licenses/by/4.0/

We thank all three reviewers for their constructive and insightful comments. Our responses are in blue below.

Referees' comments:

Referee #1 (Remarks to the Author):

It has been proposed for some years that tau assemblies, seen in Alzheimer's disease and a range of tauopathies, may act like prions and there is an extensive literature showing that tau assemblies can seed and spread in animal models following experimental inoculation with tissue extracts from human patients. In the current manuscript, they focus on the strain phenomenon which is central to prion pathobiology where structurally distinct prion protein fibrillar assemblies relate to distinct clinicopathological phenotypes in inoculated animals which are stable on serial passage.

They intracerebrally inoculated groups of wild type mice with brain extracts from patients with either AD or CBD, distinct neurodegenerative diseases both associated with tauopathy. They show distinct patterns of pathology in these mice (and different rates of its development) which associate with distinct tau amyloid structures indistinguishable for those in the human brains. They argue that tau filaments propagate by templated seeding akin to prion propagation and that the mouse provides a good model to study the mechanisms by which distinct tau folds produce specific patterns of neuropathology.

This is an important and insightful manuscript from distinguished authors which provides significant further evidence for the importance of prion-like mechanisms in the common neurodegenerative diseases. However, I do have a few queries and suggestions for further improving the manuscript:

1. ED Table 2 shows the experimental design. It is not clear however whether the time course study has 5 mice at each time point or 5 mice in total. There are no further details of these experiments in the methods. n = 1 for the early time points would raise concerns about statistical validity so I am assuming it is n = 5. The authors need to clarify and amend ED Table 2 to make group sizes clear.

Seven mice were used for immunohistochemistry at end-stage (we revised Extended Data Table 2 and inserted a new Extended Data Table 3 with more details about the mice), but only a single mouse was used for each time point in the time-course experiment. No staining for human tau was observed. Our main motivation for the time-course experiment was to exclude the possibility that the extracted filaments at end-stage were the injected human tau seeds. The observations that sarkosyl-insoluble tau and tau pathology by immunohistochemistry disappear after 1 week and re-appear in a time-dependent manner (in addition to the data presented in Figure 2) confirm that the filaments from which we solved the cryo-EM structures were the product of seeded aggregation of mouse tau.

That said, the point of statistical validity for claims of differences in timing is well taken. Therefore, we have removed the statement that seeded aggregation with AD filaments precedes that of seeding with CBD filaments from the manuscript, and we have moved Figure 3 in the original paper to the new Extended Data Figure 4. The revised main text now reads:

*“To further eliminate the possibility that the injected human tau filaments remained intact during the seeding experiments, we performed a time-course analysis of the injected seeds using histological staining, electron microscopy and Western blotting. The human tau seeds were visible by staining immediately after injection, but they could no longer be detected after one week. AT8, pFTAA and Gallyas-Braak silver staining showed tau inclusions in mice injected with CBD seeds at 1mpi and in mice injected with AD seeds at 3mpi; in both AD and CBD-injected mice tau inclusions had increased in number by 6 mpi and 9 mpi (**Extended Data Figure 4**). Clearance of the injected human seeds and time-dependent increase in mouse tau inclusions was confirmed by negative-stain electron microscopy and Western blotting using anti-Tau C, mTau and AT8 antibodies (**Extended Data Figure 5**).”*

2. There is some lack of clarity regarding the investigations in mice, specifically what proportion of inoculated mice showed what pathology. A conventional transmission table with a column for pathological findings would be very helpful.

We have revised Extended Data Table 2 and inserted a new Extended Data Table 3 with the pathological findings in the different mice.

3. The cryo-EM data look convincing (as would be expected from these authors), and I assume the preliminary PDB validation reports that were submitted will be updated with the official PDB validation reports with the revised manuscript.

The maps and models have now been released by the EMDB/PDB, and all validation information is publicly available.

4. If tau strains are transmissible to conventional mice, then we should assume they are probably transmissible to humans if similarly inoculated. The circumstances for iatrogenic or laboratory transmission to humans will hopefully be very limited but nonetheless I think the authors should mention this concern in their discussion, not least with respect to laboratory workers handing highly purified seeds prepared for cryo-EM, presumably of high titre. Iatrogenic transmission of AD has been reported (Banerjee et al Nature Medicine 2024) and this could also be cited in this regard.

We included the following sentence in the Discussion:

“Prion diseases are infectious and can be transmitted between individuals, whereas inter-individual transmission of tauopathies has not been reported (although iatrogenic Alzheimer's disease has been reported in recipients of cadaveric pituitary-derived growth hormone that contained amyloid-beta seeds [36]).”

5. The authors argue that tau seed structure influences both cellular targeting and pathological morphology in the inoculated mice and indeed that they have demonstrated that wild type mice are a suitable model to study the molecular mechanisms by which the distinct tau filament structures drive disease-specific pathology. This may be the case but I would advise some caution here given what we understand about prion pathogenesis in mice. In scrapie prion infected mice two mechanistically distinct phases are described. Prion propagation rises exponentially early in the incubation period and prion seed titre reaches a plateau well before

neuropathology develops (see Sandberg et al Nature 2011 and Nat Comms 2014). However, as these authors note and are seeking to reproduce with tau seeds, distinct prions strains are associated with distinct patterns of neuropathology. This suggests that the neuropathology actually relates to the secondary development of neurotoxic species with selective vulnerability to neurotoxic processes rather than the distinct seeds selectively propagating and targeting different cell types or brain regions. This is beyond the scope of the current manuscript but would be worth mentioning in the discussion.

Our results show that different tau folds induce distinct cellular inclusions: CBD in glia and AD in neurons, supporting the conclusion that the seed structure contributes to disease-specific pathology. Downstream effects of the seeded aggregation of tau in these cells are not well understood, and the mouse does not develop a clear phenotype. We agree with the reviewer that the unresolved relative contributions of propagation (by templated seeding), secondary toxic species, and selective cellular vulnerability are important topics of future research, which we also plan to study.

We have added the following reference to the Sandberg paper to the Discussion:

"It thus remains unclear whether tau seed propagation and downstream toxicity occur in two separate phases, as has been suggested for prions [38]."

6. Finally, I should note that conventionally, characterisation of prion strains involves serial passage in a host species, not simply the primary passage described here. Clearly such serial passage is very time consuming and I would not ask the authors to do this with respect to the current manuscript but this should be noted as a limitation in the discussion.

We agree. These experiments will be the subject of future studies. We have added the following sentence to the Discussion:

"Future serial passage experiments will provide a complete characterisation of disease-specific tau strains, and the use of seeds assembled from recombinant protein [24] will narrow down the molecular species of seeding to tau filaments alone."

Referee #2 (Remarks to the Author):

The authors report that the intracerebral injection of tau fibrils into wild-type mice induces tau aggregates that adopt the same structure as the injected fibrils. Specifically, mice injected with AD-derived Tau seeds develop pathology predominantly confined to neuronal cell bodies and neurites. In contrast, injection of CBD-derived Tau results in Tau accumulation in both neurons and glial cells, including the formation of coiled bodies and plaque-like astrocytic inclusions.

The manuscript is clearly written, logically organised and easy to follow. The experiments are carefully executed and the conclusions are well supported by the data.

However, the majority of the data (Figures 1–3) reiterates findings that have been reported previously (e.g. reference PMID: 29054878, and others cited in the manuscript), particularly the concept that exogenous tau fibrils act as seeds that template the propagation of their

specific conformations in vivo. As such, the central premise of templated Tau propagation is not novel.

The main and only advance of the present study lies in the cryo-EM characterisation of the induced (templated) tau fibrils formed (Figure 4). Demonstrating that these induced (mouse) fibrils faithfully recapitulate the structure of the injected (human) seeds is an important technical and conceptual contribution as it strengthens the validity of mouse models for studying strain-specific human tau propagation.

Nevertheless, this advance may be of greater interest to a more specialised audience. It is less clear whether the findings reach the level of conceptual novelty and general interest required for publication in a widely read journal such as Nature.

We agree with the reviewer that the main advance lies in the observation that both Alzheimer and CBD tau filaments can be recapitulated by templated seeding in the mouse brain (Figure 3 in the revised manuscript). We believe these results to be of general interest, as this is the first time that correct templated seeding of amyloid filaments from human brains has been achieved in any organism. Thereby, we establish a central pillar of the prion hypothesis for these diseases, that strains retain their structural identity upon transmission.

.

The authors rely primarily on Olig2 staining, which is not sufficient to fully support their claims regarding cell-type specificity.

In the new Extended Data Figure 2, we used a NeuN antibody to identify nerve cells, a GFAP antibody for astrocytes and an Olig2 antibody for oligodendrocytes. Antibody AT8 was used to label tau inclusions. AT8 and GFAP co-labelled nerve cells and their processes 9 mpi of AD and CBD seeds. Astrocytic plaques were labelled by AT8 and GFAP in CBD seed-injected mice, whereas coiled bodies were labelled by Olig2 and AT8 in CBD seed-injected mice. No co-localisation of AT8 staining with either GFAP or Olig2 staining was observed in AD seed-injected mice.

Referee #3 (Remarks to the Author):

Lövestam and coworkers set out to fundamentally probe the link between the structural fidelity of tau fibril templating in vivo and the pathophysiological consequences of tau aggregates being introduced into mice as a prion strain. The authors consider the tau strain to be an aggregate with a distinct fibril structure. That structure is known at the outset of the study and is specifically identified as that of tau fibrils extracted from the frontal cortex of humans afflicted with AD or CBD. In comparing the effect of injecting either strain into a mouse brain, the authors further delineate potential differences in strain transmission on the basis of fibril structure.

These experiments are in line with recently published work describing the propagation of alpha synuclein fibril seeds in mice, where in vivo templating that largely retained fibril structure was also demonstrated. That type of behavior for alpha synuclein 'strains' was to some extent anticipated by the experiments of Prusiner and co workers, and other groups using transgenic

mice to potentiate seeding and limit the species barrier. As the authors well document, many experiments have shown analogous results for tau with transgenic or wild type mice. Advancing past those studies, the current work clearly establishes the faithful templating of endogenous tau by tau fibril isolates derived from patients and injected into the brains of mice. In that regard, the study is noteworthy and stands out as a resource for the understanding the behavior of tau and other amyloid proteins as prions.

While the work is sufficiently detailed and rigorous to support its conclusions, it brings the following thoughts and questions to mind, which are worth the authors' consideration:

A question is stirred here regarding the protein-only nature of prions. That elegant hypothesis dictates that a protein aggregate acting as a template or seed is sufficient to engender disease by itself. The experiments at hand approximate such an aggregate with fibril seeds extracted from diseased patient brains, but cannot fully account for the role of associated proteins, co-factors or other molecules present in the same fibril fractions. Unlike other studies where recombinant fibrils are injected into mouse brains to cause disease, the presence of unknown agents coexisting with the injected tau seeds introduces a degree of uncertainty into the precise definition of these strains. It may be further complicated by the role played by other tau aggregates (oligomers, minor polymorphs, etc) that may also be present and influence seeding. Are truly pure, monomorphic recombinant fibrils that adopt the same fold sufficient to cause the same strain pathophysiology? Separately, proteomics and/or metabolomics analyses of the two injected strains could be informative in clarifying this point.

It is only known how to assemble full-length recombinant tau into Alzheimer PHFs; the assembly conditions to achieve CBD filaments from recombinant tau are still elusive. Kasen et al observed that injection of PHFs assembled from purified, full-length recombinant tau with 12 phosphomimetic mutations (PAD12 tau) into the brains of wildtype and human tau knock-in mice recapitulated the seeding activity of AD brain-derived tau filaments (PMID: 41107248). These results indicate that associated proteins, co-factors or other molecules are not required to induce tau pathology in the mouse brain. The injected material for the experiments by Kasen et al was confirmed to be structural homogeneous by cryo-EM analysis, which is sensitive to minority species amounting to ~1% of the filaments.

We have modified the following sentence that cites the study by Kasen et al in the Introduction:

"The injection of filaments assembled from recombinant tau into the brains of wildtype mice and of mice expressing all six human brain tau isoforms confirmed that tau filaments are the seeding species and showed that filaments with the AD-specific fold seed better than filaments with folds that have not been observed in human brains [24]."

The finding that AD and CBD seeds induce distinct patterns of pathology in the mouse brain is noteworthy and perhaps expected. However, the true extent of those differences is difficult to assess. For example, it is not clear from the histological evidence provided whether other aggregated tau species may be present but undetected by the antibodies used.

Given the limited resolution of light microscopy, and the limited structural specificity of anti-tau antibodies in immunohistochemistry, histological staining indeed provides too little information about the structural nature of tau species in the brain. However, by cryo-EM, the sarkosyl-

insoluble fractions were structurally homogeneous for PHFs in the AD-injected mice and contained mostly type-I CBD filaments for the CBD-injected mice, with a small fraction of that we believe are type-II CBD filaments.

With regard to GT-38 binding, whose binding site is cleverly suggested to reside in tau R2: The authors note that "the epitope in R2 is accessible for GT-38 binding in the filaments from mouse brains injected with AD tau seeds, but that the same epitope is not accessible in the filaments from mice injected with CBD tau seeds, because it forms part of the ordered filament core." That assumption may be true in the case of GT-38 binding to CBD tau fibrils, but may not generally hold for other tau antibodies, since they could potentially bind motifs in ordered fibril cores. In short, a clearer picture of the GT-38 epitope is be needed to fully support the claims.

The conclusion that GT-38 binds to R2 in tau is supported primarily by the *in vitro* results against filaments with the AD or CTE folds. GT-38 does not bind to filaments consisting of only 3R tau, but GT-38 does bind to filaments that are made of 4R tau, or to filaments that are made of a mixture of 3R and 4R tau.

Antibodies with epitopes inside the ordered core of filaments can sometimes still give positive staining by immunohistochemistry. Possibly, their epitopes become available for antibody binding after tissue fixation. Therefore, epitope mapping by immunohistochemistry is not reliable.

To address the reviewer's reservation, we have toned down our statements on the epitope of GT-38, which now read:

"These findings suggest that the epitope of the GT-38 antibody lies within the second microtubule-binding repeat (R2), which is absent in 3R tau. Because mice express exclusively 4R tau in the adult brain, these results are consistent with the hypothesis that the epitope in R2 is accessible for GT-38 binding in the filaments from mouse brains injected with AD tau seeds, but that the same epitope is not accessible in the filaments from mice injected with CBD tau seeds, because it forms part of the ordered filament core."

The authors claim that their ~10 Å structure of fibrils isolated form mice injected with the CBD strain suggested these were "identical to type II CBD filaments." However, this is difficult to confirm based no the relatively low resolution data being considered.

We have rephrased this as: *"A reconstruction of the doublets, to a resolution of 10 Å, suggested that they may be type II CBD filaments."*

The lag period described by the authors between the disappearance of exogenous seeds and the detection of nascent tau inclusions is intriguing, particularly given the differences observed in its duration in mice seeded with AD or CBD strains. How do the authors rationalize this discrepancy? Perhaps these two trains face different species barriers?

In response to reviewer #1, we have moved the time-course experiment to the Extended Data, and removed statements claiming seeding with CBD filaments precedes seeding with AD filaments.

In any case, we don't expect the two strains to face different species barriers, since the mouse and human tau sequences are identical for the entire ordered cores of both the AD and CBD folds. In the point below, the reviewer suggests this may be due to differences in the tau sequence outside the ordered core. We consider it more likely that the two different filaments interact with different cellular components, leading to different cell types being affected at possibly different timings. However, detailed mechanistic studies will be required to provide a definite answer to this question. Our data shows that the mouse brain may be an appealing model system for such studies.

The evidence for a potential absence of a species barrier in the propagation of human tau strains within wild type mice is particularly noteworthy. As noted by the authors, this may well be rationalized by the similarity in human and mouse tau sequences present within the tau fibril cores in question. However, the impact of sequence differences outside the cores cannot be dismissed and could be the reason behind the lag period observed in nascent tau aggregate formation, particularly for the AD tau strain. It is also noteworthy that the reconstructions of mouse tau fibrils generally lack some of the extraneous, often unmodeled densities observed for patient derived tau fibrils. A comparison of these features could be informative and brings questions about whether the differences are due to the challenges of templating, a species barrier in their retention, or something else entirely?

The reconstructions of both the AD and the CBD filaments of mouse tau contain similar extra densities as we have previously observed for filaments from human brains. To show this, we have added new cross-section images to Extended Data Figure 6 (panels D and H).

Since the authors discuss differences in disease onset after challenge with various prion strains, it would be relevant to consider serial passaging of strain isolates in wild type mice, which may further inform on the impact of a potential species barrier and on strain adaptation to a mouse background.

We agree that this is an interesting experiment, which we will consider for our future studies. We have added the following sentence to the Discussion:

"Future serial passage experiments will provide a complete characterisation of disease-specific tau strains."

Minor notes:

Figure 4B could benefit from further annotation, e.g. some residue numbering.

We have added residue numbers to Figure 4B.

Figure 4C may be more informative if the reference chain was colored by RMSD, more easily highlighting the regions of disagreement.

We have replaced the worm-like backbone trace with a clearer stick representation for the Calpha atoms to better show the differences between the two models. Moreover, we now report the backbone root mean square deviations of the AD and CBD structures in the Figure

legend. At values below 1 Å, both the AD and the CBD structures from mouse tau are deemed identical to the human ones.

The species being noted in Extended data figure 3 would be more clear if insets were included with higher magnification views.

We have added insets with higher magnification views to show the small filaments in Extended data figure 3D.

Our responses below are in blue.

Referee #1 (Remarks to the Author):

The revised manuscript is significantly improved.

The authors now acknowledge the n=1 limitation of their mouse time course study in ED Table 2 and in the text.

A new transmission table (ED Table 3) is now provided to describe pathology findings in the mice which is helpful.

Regarding the potential for transmission of tau pathology to humans, additional text and a citation has been added regarding the recent recognition of iatrogenic AD. However, their original statement that "prion diseases are infectious and can be transmitted between individuals, whereas inter-individual transmission of tauopathies has not been reported" remains unchanged. Human prion diseases are not transmitted between individuals in normal circumstances, transmission of CJD requires inoculation with human prions during medical or surgical procedures. These iatrogenic CJD cases are very rare and account for only around 1% of CJD. The argument of this paper is that there are strains of tau amyloid that can act as prions and induce strain-specific neuropathology in inoculated mice. If correct, surely it must follow that such human tau strains would also pose a risk to humans, who after all express homologous tau proteins, if there were similarly inoculated. The concern is not of inter-individual transmission as such, but that laboratory workers intentionally purifying tau amyloid seeds for structural or other studies could be at risk from accidental inoculation. I think a clearer but measured statement of potential risk, which logically follows from their own data, is warranted.

The risk of human tau strains and those of prion protein to humans is likely to be different. Besides intracerebral injection, so far there is no identified risk of peripheral injections (or even ingestion) of tau strains. We have added the following sentence to the Discussion to reflect this:

"It remains to be seen if the peripheral administration of tau seeds can cause neurodegeneration."

The authors have added relevant text in response to my comments 5 and 6.

Referee #3 (Remarks to the Author):

The authors have appropriately addressed the review comments and present an improved manuscript suitable for publication.

My only two remaining comments are:

1. A suggestion to the authors to be quantitative in their comparisons of structures by listing their very low alpha carbon RMSD values, e.g. when stating: "This structure was identical to the Alzheimer fold from AD brains".

We have included the rmsd values in the main text when we mention the structures are identical to those of the seeds.

2. A suggestion that the authors consider the process of tau fibril clearance in their discussion paragraph

on filament assembly. Their results show that "The human tau seeds were visible by staining immediately after injection, but they could no longer be detected after one week.", suggesting that templating and clearance of fibrils co-occur in cells?

We have added the following sentence to the Discussion:
“During this stage, the human tau seeds disappear progressively, indicating that faithful templating and clearing of tau seeds are co-occurring in brain cells.”
